# All-Purpose Containers? Lipid-Binding Protein – Drug Interactions

**DOI:** 10.1371/journal.pone.0132096

**Published:** 2015-07-13

**Authors:** Tiziana Beringhelli, Elisabetta Gianazza, Daniela Maggioni, Sandra Scanu, Chiara Parravicini, Cristina Sensi, Hugo L. Monaco, Ivano Eberini

**Affiliations:** 1 Dipartimento di Chimica, via Golgi 19, I-20133 Milano, Italy; 2 Dipartimento di Scienze Farmacologiche e Biomolecolari, Università degli Studi di Milano, Gruppo di Studio per la Proteomica e la Struttura delle Proteine, Sezione di Scienze Farmacologiche, via Balzaretti 9, I-20133 Milano, Italy; 3 Dipartimento di Scienze Farmacologiche e Biomolecolari, Università degli Studi di Milano, Laboratorio di Biochimica e Biofisica Computazionale, Sezione di Biochimica, Biofisica, Fisiologia ed Immunopatologia, via Trentacoste, 2, I-20134 Milano, Italy; 4 Università degli Studi di Verona, Dipartimento di Biotecnologie, Ca' Vignal 1, strada Le Grazie 15, I-37134 Verona, Italy; University of Copenhagen, DENMARK

## Abstract

The combined use of *in vitro* (^19^F-NMR) and *in silico* (molecular docking) procedures demonstrates the affinity of a number of human calycins (lipid-binding proteins from ileum, liver, heart, adipose tissue and epidermis, and retinol-binding protein from intestine) for different drugs (mainly steroids and vastatins). Comparative evaluations on the complexes outline some of the features relevant for interaction (non-polar character of the drugs; amino acids and water molecules in the protein calyx most often involved in binding). Dissociation constants (K_i_) for drugs typically lie in the same range as K_i_ for natural ligands; in most instances (different proteins and docking conditions), vastatins are the strongest interactors, with atorvastatin ranking top in half of the cases. The affinity of some calycins for some of the vastatins is in the order of magnitude of the drug C_max_ after systemic administration in humans. The possible biological implications of this feature are discussed in connection with drug delivery parameters (route of administration, binding to carrier proteins, distribution to, and accumulation in, human tissues).

## Introduction

Carrier proteins in plasma are able to bind a number of exogenous compounds—including drugs—with close to distant resemblance to the endogenous ligands [1, 2]. This interaction, which mainly involves albumin [3] and α_1_-acid glycoprotein/orosomucoid [4], features low selectivity but high capacity (large amounts of heterogeneous substances being bound by these abundant plasma proteins), and greatly impacts on the pharmacokinetic and pharmacodynamic profile of the therapeutic agents. Plasma protein level variations with age (with the extreme case of the rapidly changing concentrations in newborns) [5] and between sexes [6, 7] do influence drug distribution. Even more so do the variations induced by disease, with albumin behaving as a negative and α_1_-acid glycoprotein/orosomucoid as a positive acute phase reactant [8]. Differences in the binding sites across species [9, 10] are confounding factors in translational medicine.

As definitely proven for albumin [11], drugs interact with the same binding sites as the endogenous ligands (for h-albumin, Sudlow’s site 1 in subdomain IIA and Sudlow’s site 2 in subdomain IIIA [12]). This is the basis for competition/displacement between endogenous and/or exogenous compounds, in health and disease (for instance, albumin *vs* fatty acids [13, 14] and uremic toxins [13, 15], and α_1_-acid glycoprotein/orosomucoid *vs* imatinib and erythromycin [16]). This is also the basis for the quest of procedures for the *in silico* estimation of drug binding to plasma proteins, and notably to albumin, as such an information is expected to contribute to the lead optimization process in early drug discovery [17, 18]. A number of albumin-based drug delivery systems are being developed [19, 20]; while some involve chemical coupling (from pro-drugs and peptide derivatives covalently bound to albumin to albumin fusion proteins), some only call for physical interaction with the binding site (high-affinity drugs are loaded into albumin nanoparticles [21], low-affinity drugs are engineered as to bind to albumin in order to extend their half-life [22]).

Interaction with exogenous compounds structurally related to the endogenous ligands may be expected as well for a number of proteins in cells whose molecular function may be described with such GO terms as *binding* and/or *transporter* (http://geneontology.org). Many of them belong to the calycin superfamily; these share a remarkable structural signature (a repeated +1 topology β-barrel) in spite of limited regions of sequence similarity [23–25] and are able to accommodate into their calyx a variety of small hydrophobic metabolites (fatty acid, steroids, bilins, retinoids). Interaction of calycins with drugs has been considered in various perspectives. The calyx architecture offers several opportunities for therapeutic applications: the most radical involves the engineering of the natural proteins to produce anticalins, antibody mimetics devised to bind specific ligands, including drugs for targeted delivery [26–29]. In a simpler option, binding to a wild-type calycin (β-lactoglobulin) preserves acid-labile drugs from exposure to the pH lows of gastric secretion and opens to their oral administration in a protein vehicle [30–32].

In contrast with the host of data available about the interaction between drugs and circulating proteins, fewer studies have been devoted to the role of intracellular proteins, which is the aim of this report.

## Materials and Methods

### c-FABPL purification

Fatty acid-binding protein from *G*. *gallus* liver (entry P80226 in http://www.uniprot.org/ database; short name = c-FABPL) was purified from chicken liver as in [33, 34]. The row animal material was obtained fresh, in batches of some kilograms, from the food market (AIA—Agricola Italiana Alimentare S.p.A., San Martino Buon Albergo (VR), Italy). The EC regulations to which the producers had to comply in their proceedings are: No 853/2004 (laying down specific hygiene rules for food of animal origin), No 854/2004 (laying down specific rules for the organisation of official controls on products of animal origin intended for human consumption) and No 1099/2009 (on the protection of animals at the time of killing). As involving food/feed items, no IACUC or ethics committee approval was required for our laboratory procedures.

After delipidation (*via* Lipidex treatment), aliquots of protein solutions in phosphate buffer (10 mM, pH 7.34) were concentrated by ultrafiltration, typically to ca. 1 mM as estimated by UV measurements (ε_280_ = 9423 M^-1^ cm^-1^).

### Ligands

The ligands for *in vitro* and *in silico* tests are listed in Table 1. Those used in NMR experiments were purchased from Sigma Aldrich and used as such. Fluvastatin, a generous gift from Novartis, was carefully desiccated under vacuum and stored under nitrogen in sealed vials.

**Table 1:** Data on the test ligands.

#	name	log P	activity
**1**	norfloxacin	-1.13	synthetic antibacterial
**2**	dexa-methasone phosphate	-0.32	synthetic glucocorticoid
**3**	5-fluoro-salicylate	-0.11	salicylate = active metabolite of aspirin, a nonsteroidal anti-inflammatory
**4**	pravastatin	1.11	synthetic antilipidemic
**5**	dexa-methasone	1.90	synthetic glucocorticoid
**6**	beta-methasone	1.90	synthetic glucocorticoid
**7**	cholate	2.11	bile acid
**8**	R-flurbiprofen	2.35	nonsteroidal anti-inflammatory
**9**	S-flurbiprofen	2.35	nonsteroidal anti-inflammatory
**10**	fluocinolone acetonide	2.37	synthetic glucocorticoid
**11**	simvastatin	2.77	synthetic antilipidemic
**12**	fluocinolone acetonide acetate	2.94	synthetic glucocorticoid
**13**	sulindac	3.03	nonsteroidal anti-inflammatory
**14**	fluvastatin	3.39	synthetic antilipidemic
**15**	cerivastatin	3.81	synthetic antilipidemic
**16**	R-fluoro-palmitate	4.17	palmitate = medium-chain fatty acid
**17**	S-fluoro-palmitate	4.17	palmitate = medium-chain fatty acid
**18**	palmitate	4.22	medium-chain fatty acid
**19**	atorvastatin	5.25	synthetic antilipidemic
**20**	cholesterol	7.39	membrane lipid

Natural and synthetic compounds are listed in order of increasing partition coefficient (logP)

### ^19^F-NMR spectroscopy

Stock solutions of poorly water-soluble ligands were prepared by dissolving weighted amounts in freshly distilled organic solvents: CH_2_Cl_2_ for norfloxacin (ca. 10 mM), flurbiprofen (ca. 15 mM), sulindac (ca. 10 mM), 5-fluorosalicylic acid (ca. 27 mM); MeOH for dexamethasone (ca. 10 mM); diethylic ether for 2-fluoropalmitate (ca. 5.5 mM). Volumes of these solutions appropriate to result in varying stoichiometric ratios to c-FABPL were transferred in 5 mm NMR tubes and the solvent was slowly evaporated in a dry nitrogen stream. Stock solutions of fluvastatin sodium salt (ca. 10 mM) and dexamethsone-21-phosphate disodium salt (ca. 35 mM) were prepared in 10 mM phosphate buffer pH 7.4 (PBS). c-FABPL/ligand binding stoichiometry was established through stepwise interaction experiments. Protein solutions were sequentially incubated with a defined stoichiometric amount (typically 0.5 eq) of the ligand and the interaction process was monitored through the acquisition of a series of ^19^F spectra. For the poorly soluble ligands, parallel control experiments were performed in the absence of c-FABPL, by monitoring their possible dissolution in PBS through the acquisition of ^19^F spectra.

For all the other experiments, c-FABPL solutions were previously incubated with the appropriate amount of ligand in the NMR tube for at least 12 h in a thermostatic bath at 298 K. Typically, {^1^H}^19^F spectra were acquired at 298 K on a Bruker DRX300 equipped with a QNP probe collecting 240–400 transients of 8 K data points over a spectral width of 14,000 Hz. The standard inversion recovery pulse sequence was used for the longitudinal relaxation time (T_1_) measurements employing 10–12 variable delays (ranging from 1 ms to 25.0 s) for each experiment and allowing for complete relaxation a delay at least five times the estimated relaxation time. T_1_ values were obtained from a non-linear three parameters fit of the experimental data. The heteronuclear Overhauser enhancement (heteronuclear n.O.e.) was estimated according to the following relationship: η = (I_d_ − I_0_)/I_0_, where I_d_ and I_0_ are the integrated intensities of ^19^F signals in spectra acquired with and without proton decoupling during the relaxation time.

### Competition experiments

Solutions of selected c-FABPL complexes in PBS were treated with stoichiometric or stepwise increasing amounts of putative competitive ligands. Appropriate volumes of stock solutions of the test ligands were added directly to the c-FABPL complex solutions (when the ligand was buffer soluble) while poorly soluble species were previously deposited through evaporation into an NMR tube in which the c-FABPL complex solution was then transferred. Reference ^19^F-NMR data were recorded before and after overnight incubation at 298 K. In some cases ^19^F spectra were also recorded during the incubation step.

### Molecular docking

All computational procedures were performed with modules and programs of the MOE suite (Molecular Operating Environment, release 2013.08, by Chemical Computing Group Inc., Montreal, QC, Canada).

**Fig 1:**
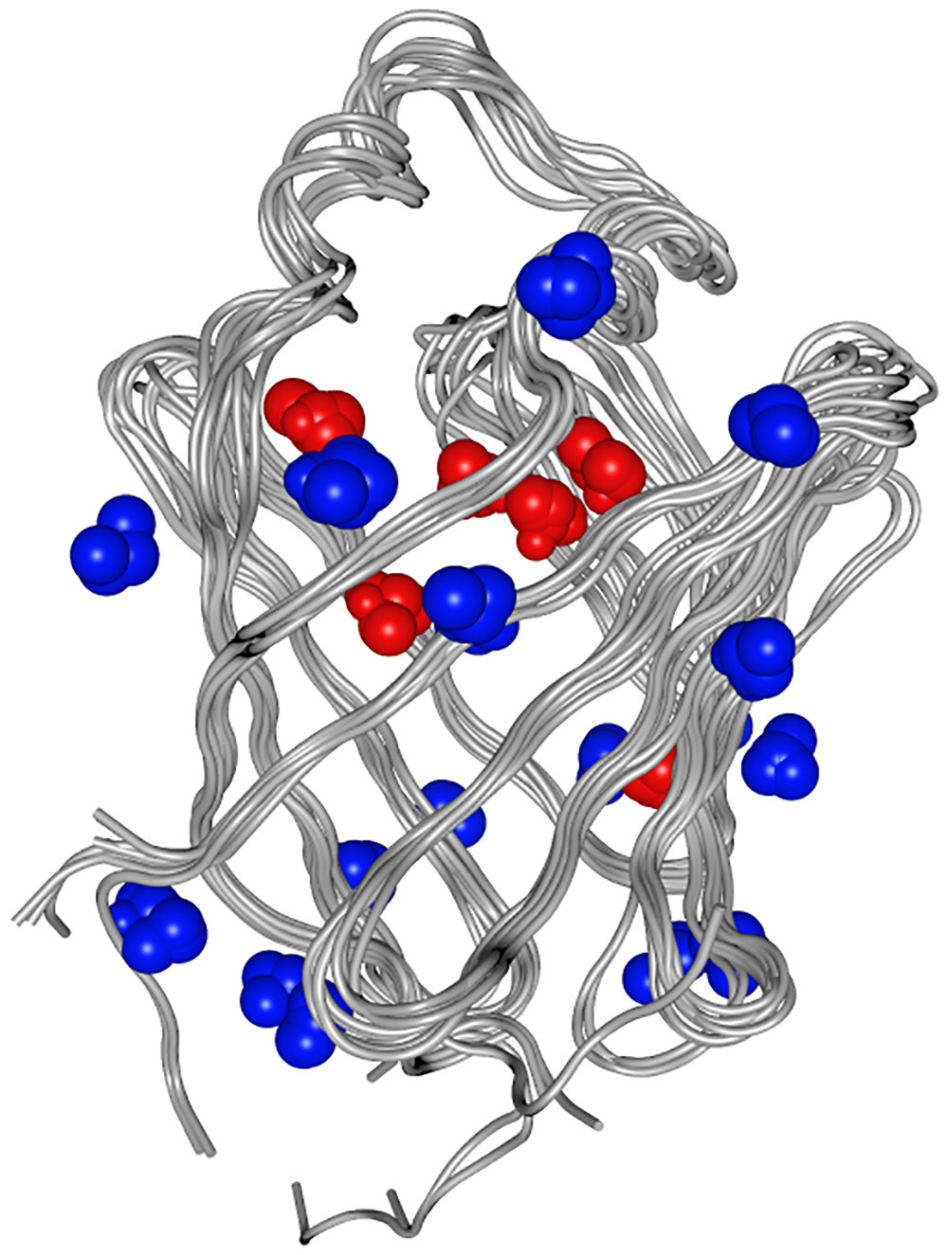
Consensus water for the 11 calycin structures in Table 2 resolved by X-ray crystallography, displayed against a line rendering of the backbone of the same proteins. Water molecules rendered in red map inside, in blue outside the calyx.

“A consensus water molecule is defined as one that is within 1 Å of another water molecule seen in at least one other structure” [35]. To identify consensus water, the 11 structures of the selected calycins that had been resolved by X-ray crystallography (Table 2, notes) were superposed after alignment of the protein sequences with T-Coffee (Tree-based Consistency Objective Function For alignment Evaluation http://www.ebi.ac.uk/Tools/msa/tcoffee/) (alignment data in S1 Fig). The identification procedure (a feature of the 2014 release of the suite, described in file:///Applications/moe2013/html/proteins/procore.htm#WaterConsensus) was repeated on the protein structures while changing the *water site contribution* parameter. A value of 6 with B-factor added to RMSD was set and produced the output in S1 Table, corresponding to the constellation of water molecules shown in Fig 1.

**Table 2:** Data on the test calycin structures^a^.

#	species	organ	short name	UniProt^b^	RCSB^c^	ligand	RCSB^c^	ligand
**I**	*G*. *gallus*	liver	c-FABPL	P80226	1TVQ^d^	*apo* form	1TW4^d^	2x cholate
**II**	*H*. *sapiens*	ileum	gastrotropin	P51161	1O1U^e^	*apo* form	1O1V^e^	1x taurocholate
**III**	*H*. *sapiens*	liver	h-FABPL	P07148	3STN^d^	*apo* form	3STK^d^	2x palmitate
**IV**	*H*. *sapiens*	heart	h-FABPH	P05413	1G5W^d^	*apo* form	2HMB^d^	1x palmitate
**V**	*H*. *sapiens*	adipose tissue	h-FABPA	P15090	3RZY^d^	*apo* form	2HNX^d^	1x palmitate
**VI**	*H*. *sapiens*	epidermis	h-FABPE	Q01469	4LKP^d^	*apo* form	1B56^d^	1x palmitate
**VII**	*H*. *sapiens*	small intestine	h-RETI	P50120	2RCQ^d^	*apo* form	2RCT^d^	1x retinol

^a^ proteins are listed in order of decreasing similarity to c-FABPL

^b^ http://www.uniprot.org/

^c^ http://www.rcsb.org/pdb/home/home.do

^d^ structure resolved by X-ray crystallography

^e^ structure resolved by NMR

The number of consensus water molecules varies between 1 and 5, depending on the protein and on the form of the protein being *apo* or *holo*. Non-consensus water molecules were (manually) removed from the pdb file of the *holo* form for each of the target proteins, and the protein structures were protonated (*3D protonation*).

The database of ligands in Table 1 was docked under default conditions to the resulting protein structures, first in the presence of consensus water then in its absence, using the Amber12:EHT force field (*docking protocol* = rigid receptor, *receptor* = receptor+solvent, or receptor atoms, *site* = ligand atoms, *placement* = triangle matcher, *rescoring-1* = London dG, *refinement* = forcefield, and *rescoring-2* = GBVI/WSA); only docking in the absence of water was feasible with gastrotropin, whose structure has been resolved by NMR. In all instances 1:1 protein/ligand complexes were simulated.

In detail, rigid molecular docking was carried out on the *holo* protein structures targeting the site of the crystallographic ligands. Poses were generated by superposition of ligand atom triplets and triplets of receptor site points, the latter *alpha sphere* centers, i.e. locations of tight packing. The 30 top-scoring non-duplicate poses were subjected to an energy minimization procedure and then re-scored according to the GBVI/WSA dG scoring function, which computes an approximate value for binding free energy.

To validate the accuracy of the above protocol in reproducing ligand binding orientation with the specific receptors and under the specific conditions used in the present investigation, we searched in our experimental set for a protein to have been crystallized both in the presence of a natural ligand and of a synthetic drug. For h-FABPA, in the RCSB Protein Data Bank (http://www.rcsb.org/pdb/home/home.do) we found 2HNX, which contains palmitate (see Table 2), and 6P6G, which contains ibuprofen. We docked both ligands back to the cognate X-ray structure: the top scoring rmsd between computed and experimental position of ligand atoms was 0.84 Å when redocking palmitate to 2HNX (S2 Fig, panel A), 0.37 Å when redocking ibuprofen to 6P6G (panel B). Docking ibuprofen to 2HNX with consensus water yielded a top scoring pose completely overlapping with the crystallographic ligand when the docked complex was superposed to 6P6G (panel C).

The interactions between ligands and proteins were summarized in the format of Protein Ligand Interaction Fingerprints (PLIF) on the basis of docking data both in the presence and in the absence of consensus water.

### *in silico* dissociation constant determination

For each ligand, the top-scoring pose (according to the GBVI/WSA dG scoring parameter) was used for computing affinity. The complexes were refined through the use of a set of specific MOE molecular mechanics procedures aimed at the relaxation of ligands in the receptor binding site. During these steps, protein side chain atoms and ligand atoms were left free to move. The dissociation constant (K_i_) was then computed through the binding free energy, as estimated *via* the GBVI/WSA dG scoring function, according to the following equation: K_i_ = e^ΔG/RT^, where R represents the gas constant and T the absolute temperature (300 K).

## Results

Our group has first purified and extensively characterized structure and function of one intracellular calycin, c-FABPL; technical approaches included X-ray crystallography, ^1^H-NMR, ^13^C-NMR, molecular docking and molecular dynamics [33, 34, 36–40].

Further working on this protein, during the present investigation we carried out *in vitro* interaction tests between purified c-FABPL and a number of fluorine-containing compounds with unlike chemical structure, as shown in Table 1; selection criteria favored drugs and drug analogs with diverse pharmacological activity, differing in molecular mass and partition coefficient. Binding was assessed by evaluating the ^19^F-NMR spectra of protein-drug mixtures. *In silico* simulations of the interaction between c-FABPL and the test ligands were performed in parallel and compared with the *in vitro* findings. The same compounds were then docked to selected human intracellular calycins to evaluate the likelihood of *in vivo* protein/drug interactions.

### *in vitro* data: c-FABPL interactions

The high sensitivity of ^19^F heteronuclear NMR proves very useful for assessing ligand binding, mainly through the measurement of the longitudinal relaxation time (T_1_) and of the heteronuclear Overhauser effect (heteronuclear n.O.e.). Indeed, sometimes only small changes are observed in the chemical shift of the ligand reporter nuclei after protein/ligand interaction. Instead, the relaxation parameters are extremely sensitive to the changes of the motional regime that occur upon binding of the ligand to a macromolecule. The increase of the correlation time results in a significant decrease of T_1_ and n.O.e., the latter eventually reaching negative values (for ^19^F, η_max_ = -1.04 in the slow motion regime *vs* η_max_ = + 0.54 in the extreme narrowing conditions).

As shown in Table 3, the relaxation parameters evaluated after the incubation with c-FABPL, in particular the negative n.O.e. values, point to the occurrence of binding between the protein and all the test ligands.

**Table 3:** ^19^F-NMR data on protein-ligand complexes.

#	ligand	δ free	δ bound	T1 free (s)	T1 bound (s)	η free	η bound	ligand/protein ratio
**1**	norfloxacin	-124.72	-124.55	0.86	0.27	0.22	-0.18	
**2**	dexamethasone phosphate	-164.43	-164.27	0.74	0.39	0.34	-0.41	
**3**	5F-salicylate	-125.08	-125.24	4.88	1.03	0.24	-0.58	
**5**	dexamethasone	-164.20	-163.78	0.81	0.28	0.41		0.5/1
			-163.90		0.32		-0.77	1/1
			-163.97		0.36			1.5/1
			-163.98		0.40		-0.46	2/1
**8–9**	flurbiprofen	-119.24	-118.90	2.0	0.54	0.19	-0.66	1/1
			-119.09		0.52		-0.57	
			-119.08		0.67		-0.29	2/1
			-119.15		0.68		-0.57	
**10**	fluocinolone acetonide	-186.45	-186.50	0.74	0.27	0.32	-0.84	
		-165.12	-165.30	0.96	0.33	0.19	-0.84	
**13**	sulindac	-113.65	-113.50	1.17	0.59	0.23	-0.46	
**14**	fluvastatin	-116.43	-116.31	2.03		0.26	-0.74	0.5/1
			-116.41				-0.7	
			-116.32				-0.54	1/1
			-116.40				-0.71	
			-116.40		0.55		-0.59	2/1
**16–17**	2F-palmitate	-194.14^a^	-180.94	2.47^a^	0.24	0.29^a^	-0.54	
			-181.10		0.23		-0.58	

^a^ free ligand data in CDCl3

**Fig 2:**
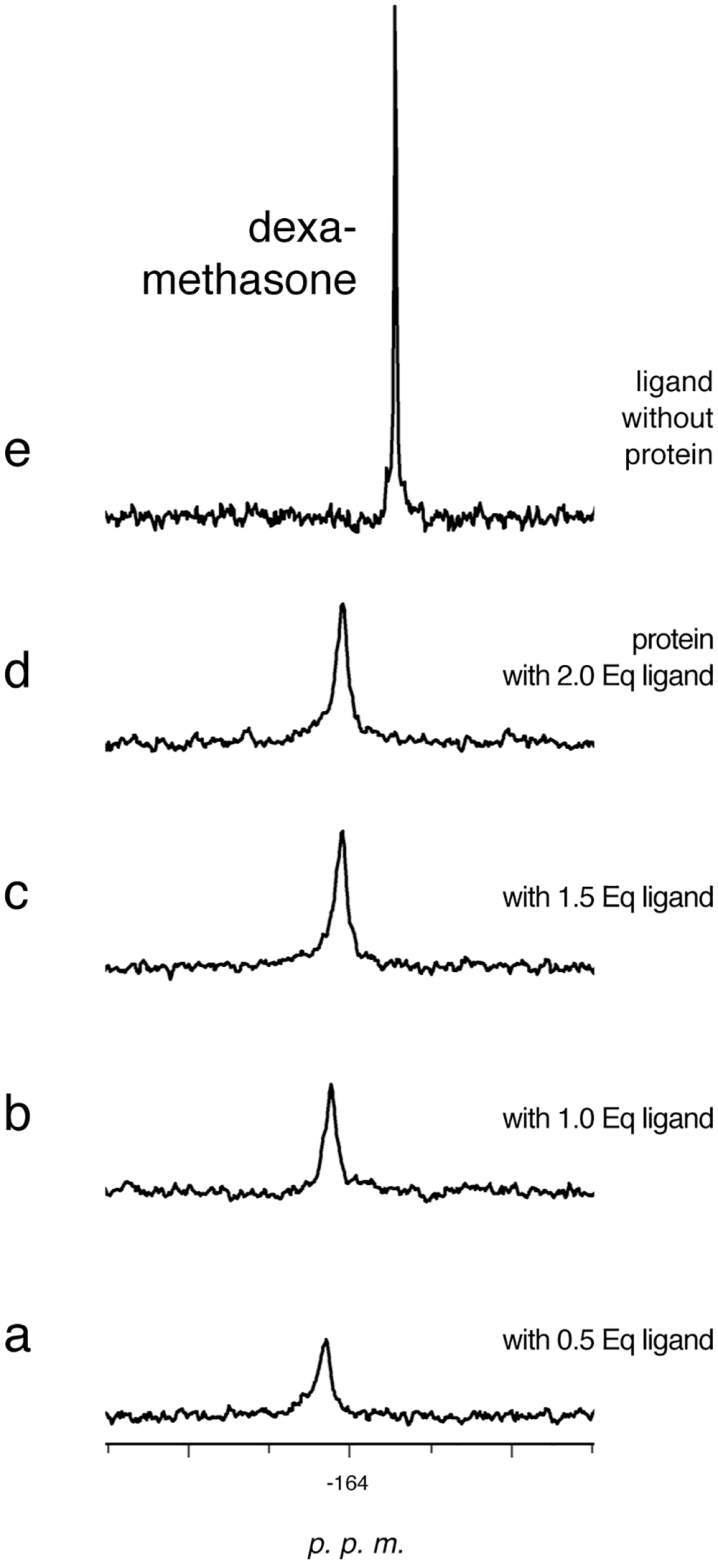
^19^F-NMR spectra recorded after the interaction of c-FABPL with increasing amounts of dexamethasone, from 0.5 to 2 equivalents (traces a-d). The spectrum of the ligand in the absence of the protein is reported in trace e. Operating conditions: phosphate buffer pH 7.4, 7 T, 298 K.

For most of the ligands a single signal is observed even in the presence of 2 equivalents of drug but the integrated intensity of this resonance does not support the binding of 2 ligand molecules. Fig 2 shows, as an example, the behavior of dexamethasone. Its buffer solubility (0.25 mM in PBS with 10% DMSO, https://www.caymanchem.com/pdfs/11015.pdf) is lower than the concentration required to saturate a 1 mM solution of the protein. However, the integrated intensity of the signal after the first step of the interaction (0.5/1 ratio) is greater than for the same amount of dexamethasone in the absence of the protein; the intensity further increases after the second step of the interaction. This indicates that c-FABPL increases dexamethasone solubility through formation of a complex (1/1 ratio). When the overall amount of the drug exceeds 1 equivalent, only a small increase of the intensity of the signal is observed, compatible with the poor solubility of the drug in buffer. With ligand exceeding 1/1 ratio, the relaxation time becomes longer and the n.O.e. less negative (Table 3). These findings support the notion that, beyond 1 eq, the single signal results from the fast exchange between the (small) number of dexamethasone molecules free in solution and those bound to the protein.

**Fig 3:**
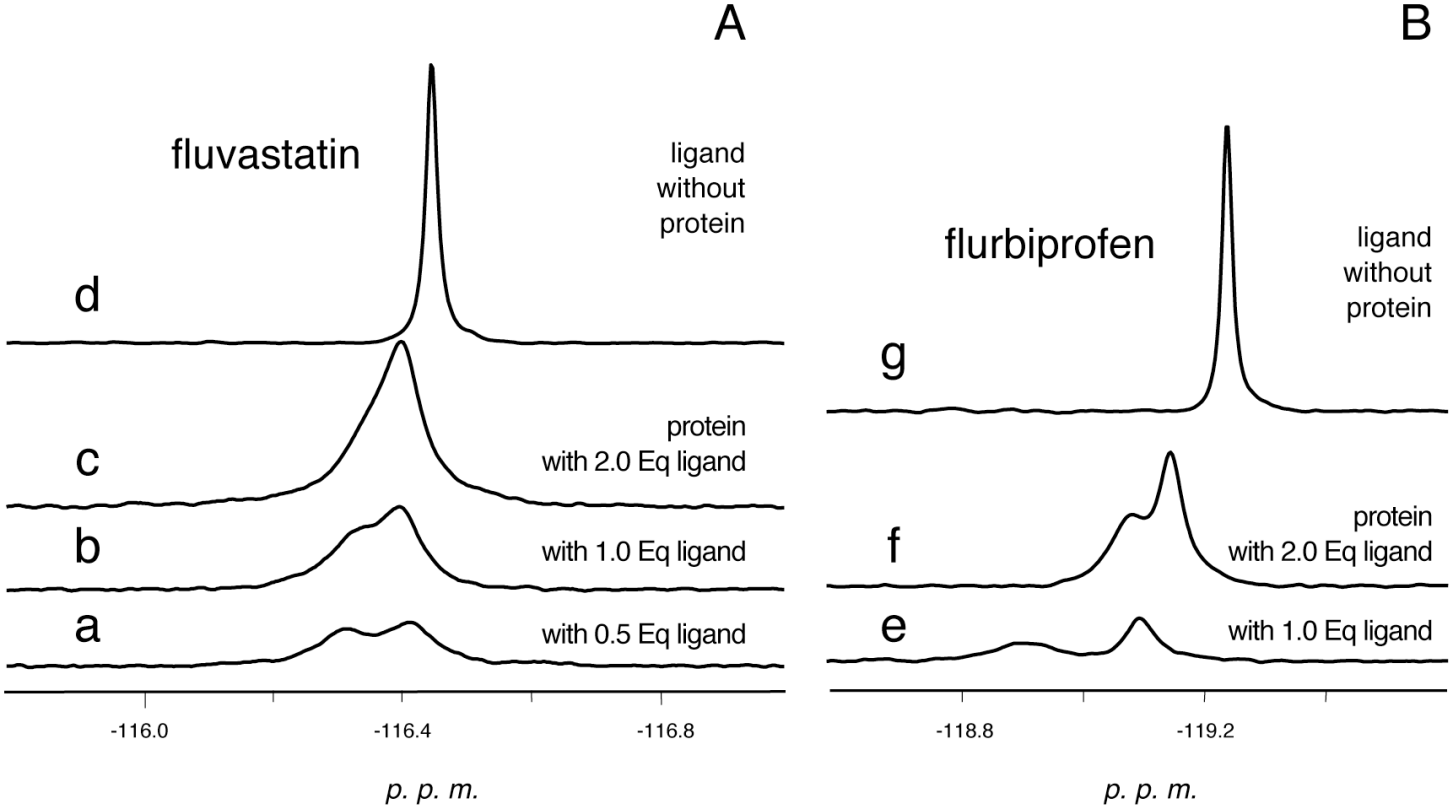
^19^F-NMR spectra recorded after the interaction of c-FABPL with increasing amounts of fluvastatin (from 0.5 to 2 equivalents; panel A traces a-c) and flurbiprofen (1 and 2 equivalents, panel B, traces e,f). The spectra of the drugs in the absence of the protein are shown in traces d (fluvastatin) and g (flurbiprofen). Operating conditions: phosphate buffer pH 7.4, 7 T, 298 K.

Instead, in the case of fluvastatin, flurbiprofen and 2-fluoropalmitate, ^19^F-NMR spectra (Fig 3 and S3 Fig) show two resonances; both their intensities increase when the protein is treated with more than 1 eq of drug (Fig 3). This latter evidence rules out the hypothesis that the two signals arise from two different conformations of each ligand in a single binding site. Previous X-ray [38] and NMR studies [41] have shown that c-FABPL can indeed accommodate in its cavity two molecules of bile acids. For both the drug/protein complexes, the two resonances have different linewidths that, as estimated through deconvolution, change on increasing the overall amount of drug: for fluvastatin 25.4 and 20.9 Hz, 25.8 and 15.1 Hz, 23.3 Hz for 0.5/1, 1/1 and 2/1 ligand/protein ratios, respectively; for flurbiprofen 26.5 and 13.3Hz, 16.4 and 8.8 Hz in the case of 1/1 and 2/1 ligand/protein ratios, respectively. Also, on increasing the overall amount of drug, the chemical shifts of both signals change and (when measurable) the n.O.e. of the broader, low-field resonance become less negative (Fig 3 and Table 3). These data suggest that two binding sites are involved, which enable different mobility for the ligand molecules: in c-FABPL the binding to the site responsible for the low-field resonance allows exchange between protein-bound ligand and free molecules in solution. In [41], ^15^N solution studies reported a similar behavior for glycocholate: the ligand in one of the two binding sites (dubbed “superficial site”) was found to exchange with free ligand in solution *(vide infra)*. The low-field resonances could therefore be attributed to drug molecules bound to the superficial site of c-FABPL. Moreover the shifts (in opposite directions) of the high field signals of fluvastatin and flurbiprofen complexes, observed on increasing the overall amount of drug, suggest also the possible occurrence of other dynamic processes involving the second site, namely exchange with the free ligand (for flurbiprofen) or exchange between the two bound ligands (for fluvastatin).

**Fig 4:**
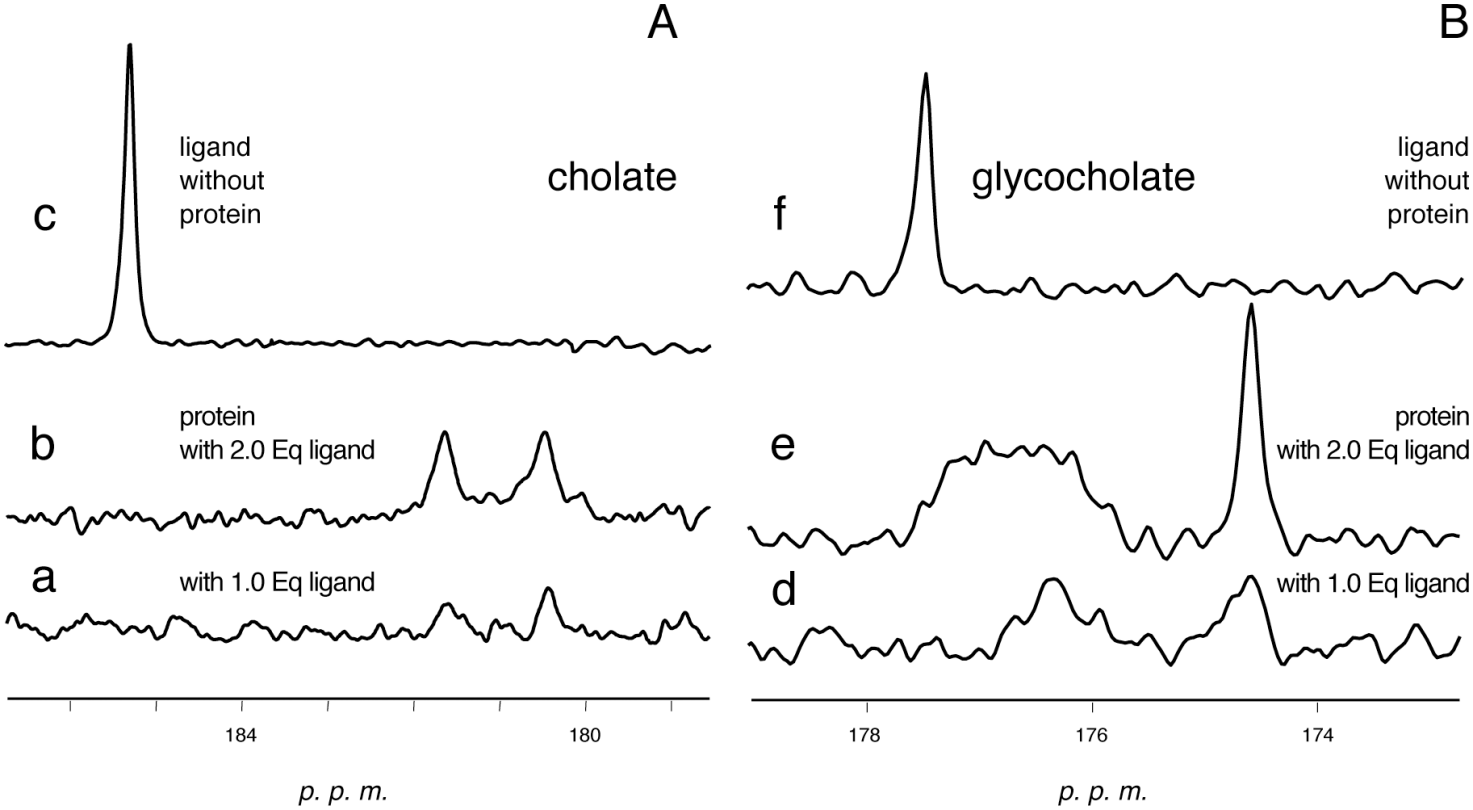
Carbonyl-carboxylic regions of the ^13^C-NMR spectra of cholic, panel A, and glycocholic acid, panel B, in the presence of c-FABPL. The spectra of the bile acids in the absence of the protein are shown in traces c and f, respectively. Operating conditions: phosphate buffer pH 7.4, 7 T, 298 K.

We then performed competition experiments between selected c-FABPL/drug complexes and cholate or/and glycocholate, two of the bile acids known to be physiological ligands for c-FABPL, using natural abundance or selectively ^13^C-enriched bile acid samples. Preliminary interaction experiments performed on the *apo* protein using selectively ^13^C-enriched ligands confirmed the presence of two different binding sites. The titration indicates two sites of comparable affinity for cholate, and of different affinity for glycocholate (Fig 4). For the latter, the broad linewidth of the low-field signal (trace e) is in line with the known occurrence of exchange with free ligand in solution [41].

**Fig 5:**
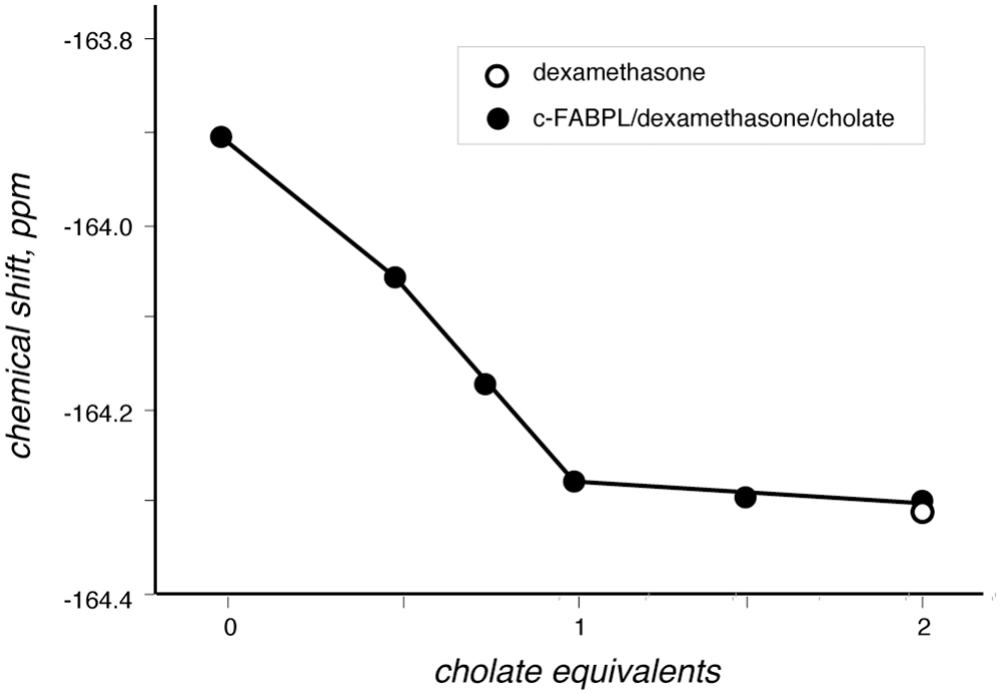
Progressive shift of the resonance of dexamethasone (solid circles) towards the values of the free ligand (empty circle) on increasing the amount of cholate in solution. Operating conditions: phosphate buffer pH 7.4, 7 T, 298 K.

**Fig 6:**
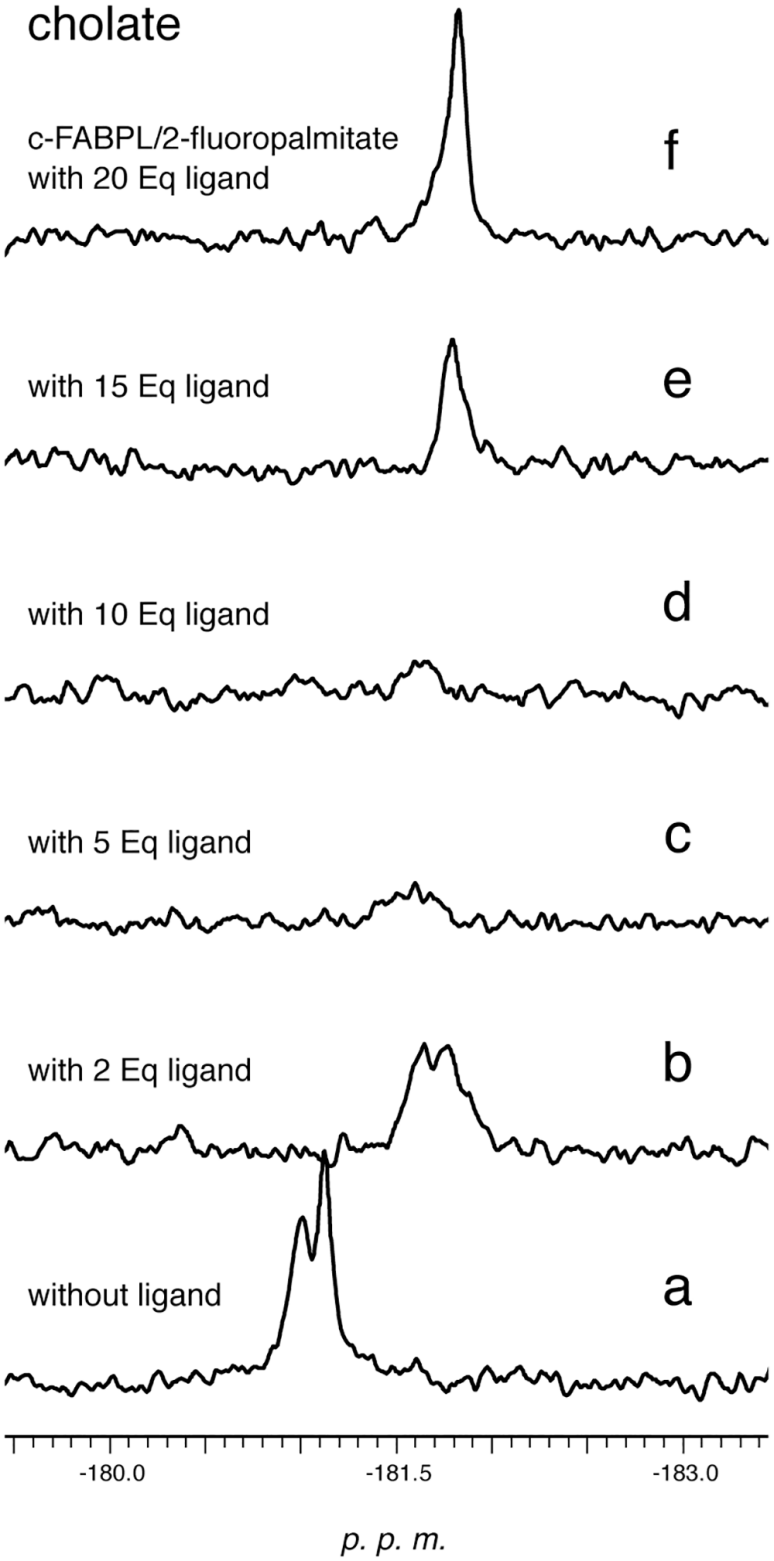
^19^F-NMR spectra of the c-FABPL/2-fluoropalmitic complex in the presence of increasing amounts of cholate (0–20 equivalents). Operating conditions: phosphate buffer pH 7.4, 7 T, 298 K.

Upon addition of cholate to the complex c-FABPL/dexamethasone, the drug was quantitatively removed from the protein at a 1:1 ratio (Fig 5), indicating a lower affinity for the drug than for cholate. Instead, in the case of the complex c-FABPL/2-fluoropalmitate (Fig 6), only using a tenfold excess of cholate the ligand was completely removed from the protein, indicating an affinity higher for the palmitate derivative than for cholate. 2-fluoropalmitate is water-insoluble, hence its ^19^F signal disappeared at first (traces c-d: the ligand displaced by cholate did precipitate) but, when cholate concentration was increased above cmc, the long-chain fatty acid was dispersed in the bile acid micelles and its signal became again visible (traces e-f).

Symmetric competition experiments between fluvastatin and cholate (that is, c-FABPL/fluvastatin complex treated with increasing amounts of cholate, and *viceversa*) proved that the two ligands bind to c-FABPL with comparable affinity. Indeed, even using an eightfold excess of the competing ligand neither the relaxation parameters nor the position of the signals did return to the values associated with the free molecule. A similar behavior was observed with glycocholate and flurbiprofen (data not shown).

### *in silico* data: c-FABPL interactions

**Fig 7:**
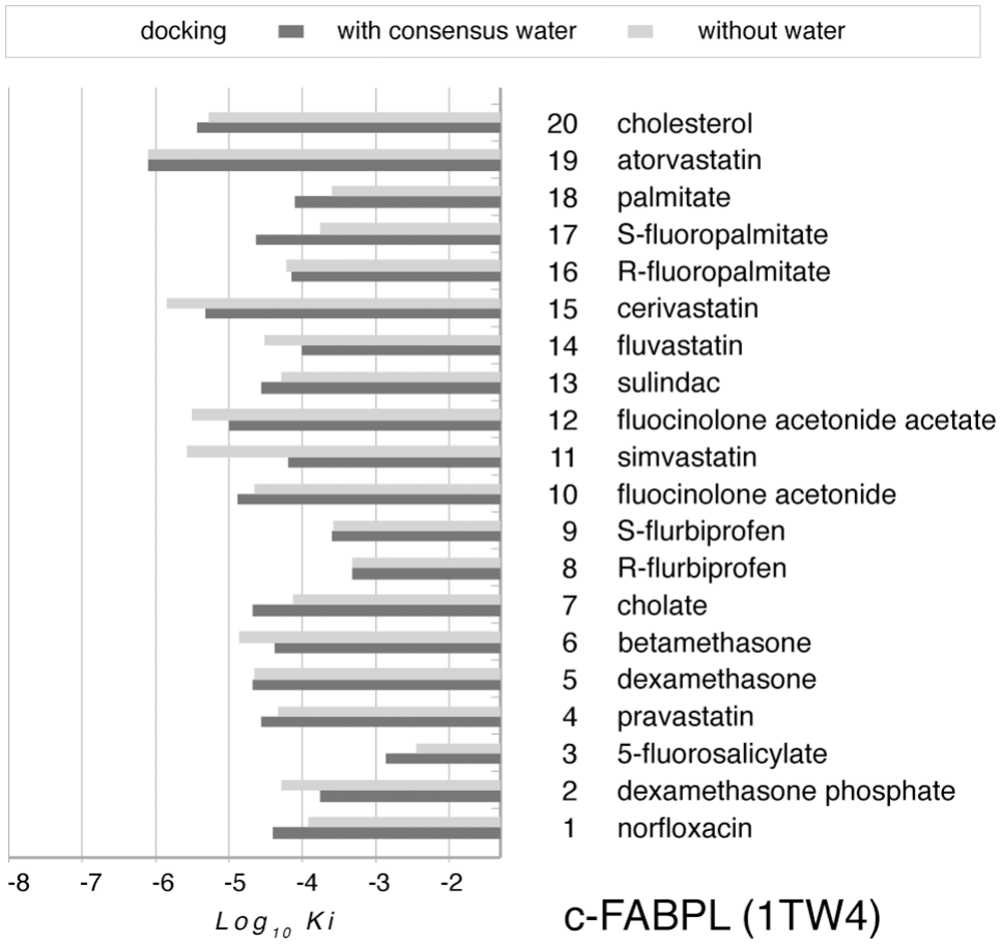
Log_10_K_i_ for the interaction of each of the test ligands (in Table 1) with c-FABPL. The *holo* structure of the protein (pdb code 1TW4) was taken as *receptor* and ligand atoms as *docking site*; the procedure was repeated in the presence (dark grey bars) and in the absence (light grey bars) of consensus water (Fig 1 and S1 Table).

The ability of *in silico* computation to hint to the same interactions observed *in vitro* was verified by docking the same chemicals to c-FABPL, with and without consensus water. The computational data are graphically rendered (as Log_10_K_i_) in Fig 7: actually, the list of the docked chemicals is slightly extended in comparison with the *in vitro* test as to include a number of vastatins. Computed Log_10_K_i_ range between -2 and -6, with half of the values (20/40) clustering at -4. Variance between results under different docking conditions appears negligible (average at -4.51 ± 0.72 in the presence of consensus water, at -4.66 ± 0.84 in the absence of water) even though, when docking is carried out in the presence of consensus water, the most frequent interaction of the ligands is with one solvent molecule (52% of the cases as recorded in PLIF; S4 Fig).

Overall, the *in vitro* and *in silico* results point to the ability of c-FABPL to bind a number of compounds with pharmacological activity and currently used in human therapy. This evidence suggested extending the investigation to human proteins homologous to the model *G*. *gallus* calycin.

### *in silico* data: human calycin interactions

c-FABPL is homolog to a number of human fatty acid and retinol/retinoic acid binding proteins, with sequence identity between 28 and 43% (not shown). To perform the *in silico* simulations under the best conditions, from the initial output of 21 entries retrieved from the RCSB Protein Data Bank, we selected six proteins for which the structure has been resolved in both the *apo* and the *holo* form; they are listed in Table 2 (the entries in order of decreasing similarity to c-FABPL) and their sequence alignment is shown in S1 Fig.

In this protein set, when structure is resolved by X-ray crystallography, calyces of both the *apo* and of the *holo* forms are noticeable for containing a substantial number of water molecules. In a previous investigation on c-FABPL we analyzed the behavior of permanent water and demonstrated its relevance in the ligand binding process [40]. On this background, as the first step of the computational part of this work we identified consensus water (S1 Table and Fig 1), to then dock the test drugs (in Table 1) to the test proteins (in Table 2) both in the presence and in the absence of these solvent molecules.

**Fig 8:**
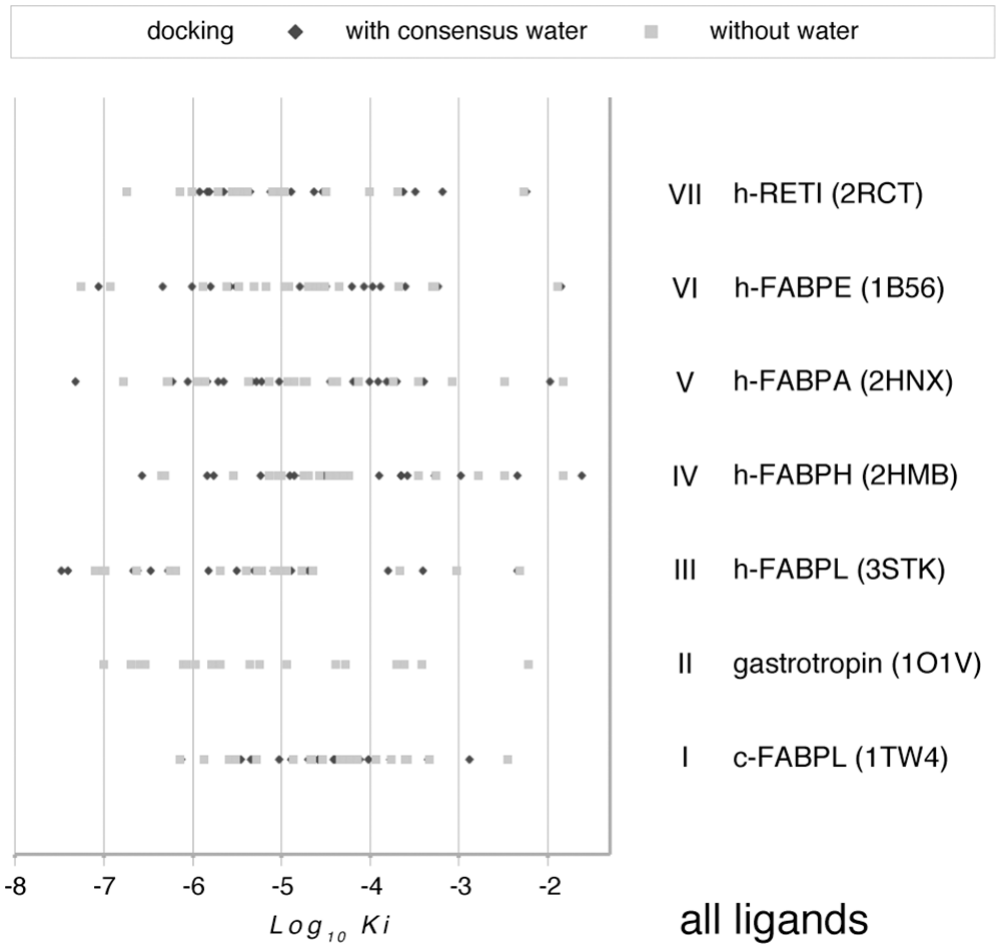
Log_10_K_i_ for the interaction of all the test ligands (in Table 1) with c-FABPL and the six human proteins with closest similarity to it (in Table 2; the caption to each data set contains the protein short name and the pdb code of the test structure). As in Fig 7, docking was repeated in the presence (dark grey diamonds) and in the absence (light grey squares) of consensus water (S1 Table) except for gastrotropin, for whose structure only NMR data are currently available.

The results from the docking procedure are compared with one another and with c-FABPL in S5 Fig; data for each protein are then grouped in Fig 8.

Log_10_K_i_ averaged for each protein across all ligands to provide an easy framework for comparison range between -4.5 and -5.4 (with SD between 0.8 and 1.3; not shown). The average affinities of the human proteins are thus equal to (with h-FABPLH), or higher than (with h-FABPA, h-FABPE, h-RETI < gastrotropin, h-FABPL), the affinity of c-FABPL for the test compounds. The proteins whose average affinities most widely diverge from the average affinity of c-FABPL are the ones with closest similarity to it. This evidence suggests that position and identity of the mutated amino acids may impact on protein function more extensively than the sheer number of mutations events.

**Fig 9:**
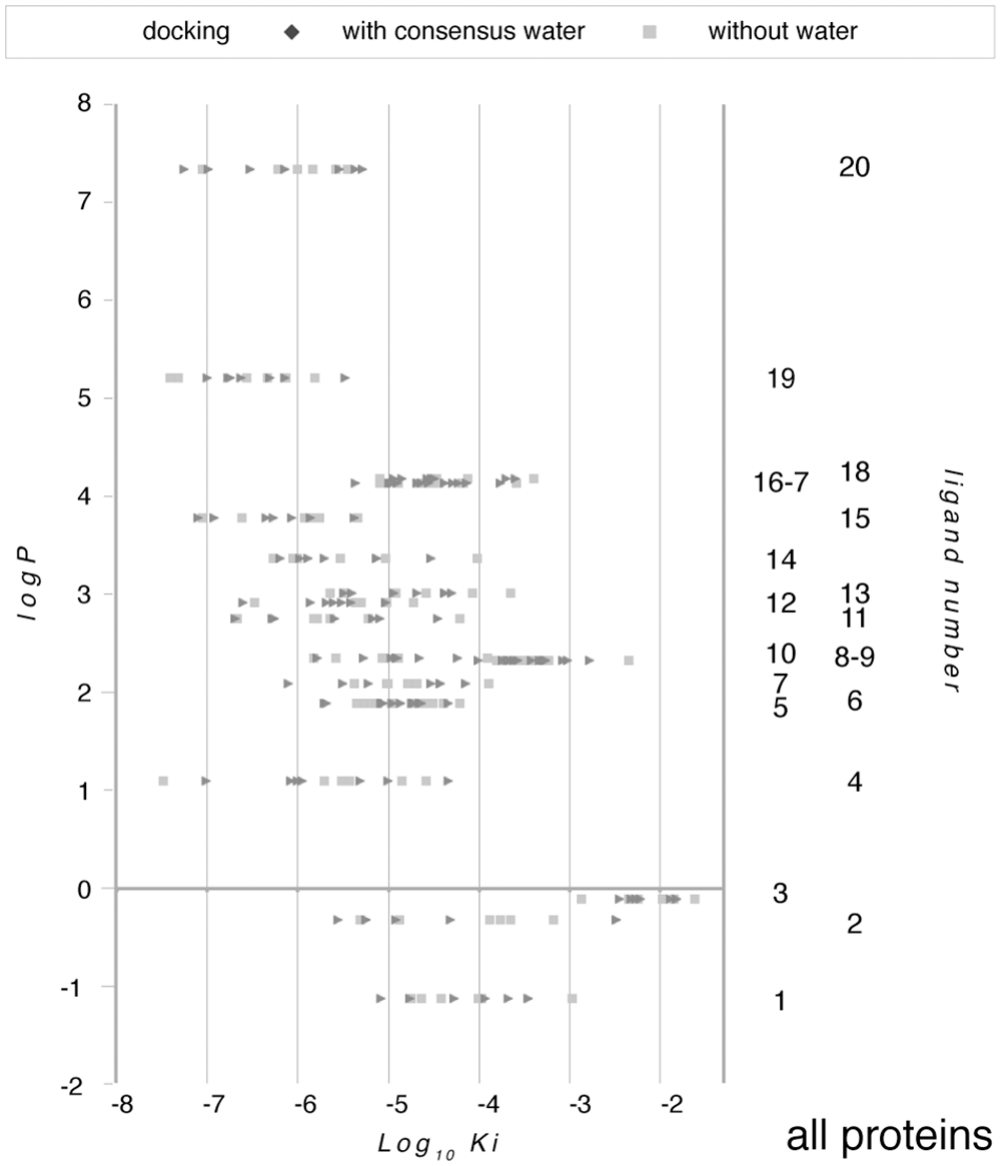
Log_10_K_i_ for the interaction of each of the test ligands (in Table 1) with the seven test calycins (in Table 2). Color coding as in Fig 7. Ligand by number as in Table 1.

The same data of Fig 8 are then grouped for each ligand in Fig 9; as in the list of Table 1, the entries are ordered according to their partition coefficient (logP; lower = polar, to higher = apolar). While there is a trend of the affinity for the test proteins to increase as the ligands become increasingly hydrophobic (Log_10_K_i_ = -0.31 logP - 4.01, for docking with consensus water; Log_10_K_i_ = -0.31 logP - 3.96, for docking in the absence of water), the statistical significance of the correlation is slight (R^2^ = 0.259 and 0.26, respectively). If the ligands are sorted on the basis of their structure, the correlation between logP and affinity appears even lower for vastatins (N = 5; Log_10_K_i_ = -0.25 logP—5.06, R^2^ = 0.16, with consensus water; Log_10_K_i_ = -0.20 logP - 5.25, R^2^ = 0.14, without water) but somewhat higher for steroids (N = 9; Log_10_K_i_ = -0.19 logP - 4.54, R^2^ = 0.31, with consensus water; Log_10_K_i_ = -0.19 logP - 4.65, R^2^ = 0.23, without water).

No correlation appears to exist between affinity and topological polar surface area (tPSA) (Log_10_K_i_ = -0.01 tPSA - 4.54, R^2^ = 0.0268; Log_10_K_i_ = -0.01 tPSA - 4.487, R^2^ = 0.04).

For ligands, a single physicochemical parameter is thus insufficient to envisage affinity as much as a single genetic parameter is for proteins, although polarity seems to play a certain role.

Indeed, in a summary of highest affinity interactions across all proteins, atorvastatin largely prevails, with 7/13 instances, followed by cerivastatin (3/13), cholesterol (2/13) and pravastatin (1/13). Except for the latter, the top-ranking ligands thus correspond to the compounds with highest logP among vastatins (2 cases) and sterols (1 case).

In order to point out which features in the test calycins are most relevant for affinity to the test ligands, we obtained PLIF reports for all proteins (S4 Fig) and selected the interactions occurring in more than 30% of cases (arbitrary cutoff). The list of such items can be read in S2 Table while their rendering is shown in S6 Fig. Color-coding based on protein lets recognize coincidence in few, seemingly crucial locations.

## Discussion

Extensive literature data support the notion that calycins are able to bind a variety of both natural and synthetic compounds. Among the latter, during focused investigations, interaction has been demonstrated between selected calycins and selected drugs. The aim of the present investigation was to test for possible interaction a number of calycins as well as a number of drugs. This transversal screening had as its starting point a series of *in vitro* assays on one calycin purified from natural sources and a panel of drugs with different chemical structure. Occurrence of protein-ligand interaction was assessed with a procedure—NMR spectroscopy—fully adequate to provide the yes/no qualitative response we were looking for at that stage but less suitable to produce quantitative data on protein-ligand affinity. To carry out the transversal screening we used instead an *in silico* approach. This was the only affordable possibility to evaluate the interaction between many proteins and many ligands. The procedures of molecular docking are robust and fully tested in molecular systems similar to those we were investigating. The specific protocol we actually used in the present investigation was validated as able to reproduce the spatial setups of a few experimental calycin-ligand structures (S2 Fig). Some of the *in silico* data turned out to closely compare with *in vitro* data obtained by other groups using different experimental approaches from ours (*vide infra*).

As detailed under Methods, the *in silico* protocol included some simplifications. 1. Docking was to *holo* forms of all the test calycins, as crystallized with a physiologically relevant natural ligand. This choice accounts for the overall rearrangement brought about by binding without fine-tuning it as a function of the ligand till the phase of protein-ligand affinity evaluation. 2. Docking was performed both in the absence of any water from the calyx and in the presence of consensus water. The latter were selected among the crystallographic water molecules in each calycin as the most likely to be relevant in the interaction. While the database of structures used to recognize such consensus molecules also included for each crystallized protein both the *apo* and the *holo* forms, the possibility that in individual cases the selection includes (one) irrelevant or excludes (one) relevant molecule cannot be entirely excluded. 3. Docking was carried out to mimic a 1:1 interaction stoichiometry. Of the *holo* forms included in our test panel, two refer to calycins crystallized with 2 molecules of natural ligand *per* protein molecule. One more of them, gastrotropin from various species, is reported to crystallize with 2 molecules of other natural ligands *per* protein molecule [42–44]. No summary knowledge about the stoichiometry of the interaction with calycins is available for the synthetic ligands hence the choice of evaluating a 1:1 complex may amount to oversimplification but the opposite choice of evaluating 1:2 complexes would imply unverified assumptions. In the experimental 1:2 complexes, binding affinity appears to be defined in part by the contribution of ligand-ligand interactions and the affinity varies from one ligand molecule to the other. With our choice we underestimate the actual affinity not only by systematically cutting to one-half the maximum number of allowed ligand binding events *per* protein molecule but also by not taking such ligand-ligand interactions into any account. While this has to be regarded as inaccuracy, in the context of the present investigation possibly underestimating affinity is less severe a bias than overestimating it.

Overall, the data presented in this report provide evidence that several synthetic compounds, in clinical use with a variety of indications, are able to bind to intracellular human calycins with affinities in the same range as their physiological ligands. In the majority of cases the most favorable interaction of the test proteins is with one of the drugs rather than with a natural compound (Fig 9 and comments therein).

**Fig 10:**
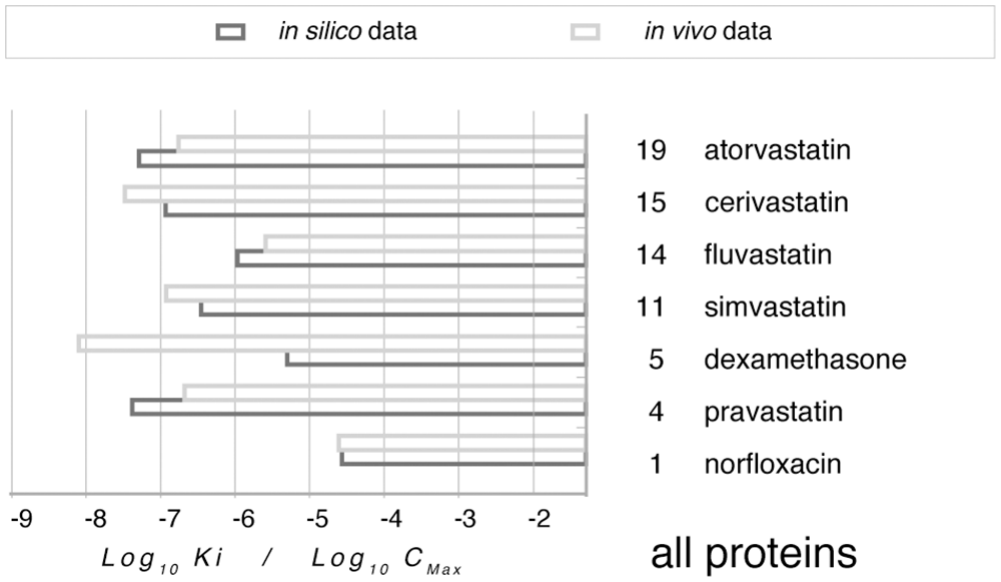
The highest affinity evaluated with any of the test calycins (Fig 9) is reported for each of the test drugs in systemic use in comparison with experimental C_max_. Data from http://www.rxlist.com/script/main/hp.asp and [45].

To verify whether such interactions may possibly have any biological relevance *in vivo*, we collected literature data on the pharmacokinetics of the test drugs. C_max_ in blood for drugs with systemic administration are marked in Fig 10 alongside the strongest interactions evaluated by molecular docking across all the human test calycins.

C_max_ is more than two orders of magnitude lower than the highest computed affinity for dexamethasone given *per os* and within one order of magnitude lower for simvastatin and cerivastatin. Conversely, peak blood concentration is comparable to the highest evaluated affinity for the antibacterial norfloxacin, and higher than this threshold for the three antilipemic medications pravastatin, fluvastatin and atorvastatin.

While *in silico* affinity data set a precondition for *in vivo* interaction, many other factors are also expected to play a role in the latter through a complex interplay. These include actual C_max_, depending on the route of administration, as well as tissue distribution, possible depot effect, binding to other extra- and intra-cellular proteins, competition between exogenous and endogenous ligands. With all of these provisos the evidence from this investigation can at most be taken as an indication for a possible *in vivo* interaction of some drugs with some calycins. However, we deem this point is worth a notice and, in perspective, a note of caution.

The human genome contains ten FABP genes (five mapping on chromosome 8 and one each on chromosomes 1, 2, 4, 5, 6) [46]. FABP expression varies across tissues both quantitatively (1–5 per cent of cytosolic proteins in hepatocytes, adipocytes and cardiomyocytes *vs* lower levels in other less active tissues) and qualitatively (FABP isoforms to serve tissue-specific functions). Proposed roles for FABPs include assimilation of dietary lipids in the intestine, targeting of liver lipids to catabolic and anabolic pathways, regulation of lipid storage and lipid-mediated gene expression in adipose tissue and macrophages, fatty acid targeting to β-oxidation pathways in muscle, and maintenance of phospholipid membranes in neural tissues [47]. If it ever occurred, interference with any of these functions would severely impact on homeostasis, from cell to organism level.

The binding properties of various FABPs for xenobiotics have already been not only recognized but indeed exploited by medicinal chemistry, with a few compounds targeting these calycins developed for diagnostic and therapeutic use. Bile acid-conjugated gadolinium chelates devised as hepatospecific contrast agents in magnetic resonance imaging (MRI) have been demonstrated to actually bind to c-FABPL [48, 49]. As involved in cytokine production and cholesterol metabolism, FABPAs are connected with inflammatory and metabolic disorders [50]; compounds with high affinity for FABPAs and high selectivity against all other FABPs thus have the potential to prevent or treat diseases such as type-2 diabetes and atherosclerosis [51–53].

The contribution of FABPLs to intestinal absorption of a number of drugs has been assessed on the recombinant rat protein using both *in vitro* and *in silico* approaches [54]. The set of test compounds in the reported investigation shares two items with our trial: acetylsalicylic acid (closely related to fluoroacetylsalicylic) and dexamethasone. For the latter, the experimental K_i_ (K_i1_ = 22.1 ± 1.5 μM, K_i2_ = 41.3 ± 3.7 μM) falls in the range of affinities we have computed for all human calycins and is very close to the figure we have evaluated for h-FABPL (10.6 μM with consensus water, 8.5 μM without water). For the former, the experimental affinity appears to be ca. 20-fold lower than for dexamethasone whereas the computed values differ approximately 100-fold.

The stoichiometry of high-affinity binding is stated to be 1:1 for all calycins except FABPLs, irrespective of the species, and for gastrotropin. FABPLs have a large solvent-accessible core (440 Å^3^) in which binding of two ligand molecules, both like and unlike in structure, has been reported. NMR (r-FABPL [55, 56]) and crystallography (h-FABPL [57]) provide evidence for rearrangements occurring in FABPL structure upon binding of the first ligand without further adjustments upon binding of the second. With two oleate molecules as ligands, a hydrogen bond between the carboxyl oxygen of the first fatty acid and the side chain NH_2_ proton of R122 plays a key role in establishing the first ligand-binding site. The K90 side chain then swivels outward to create a less crowded and more hydrophobic local environment for the second fatty acid.

**Fig 11:**
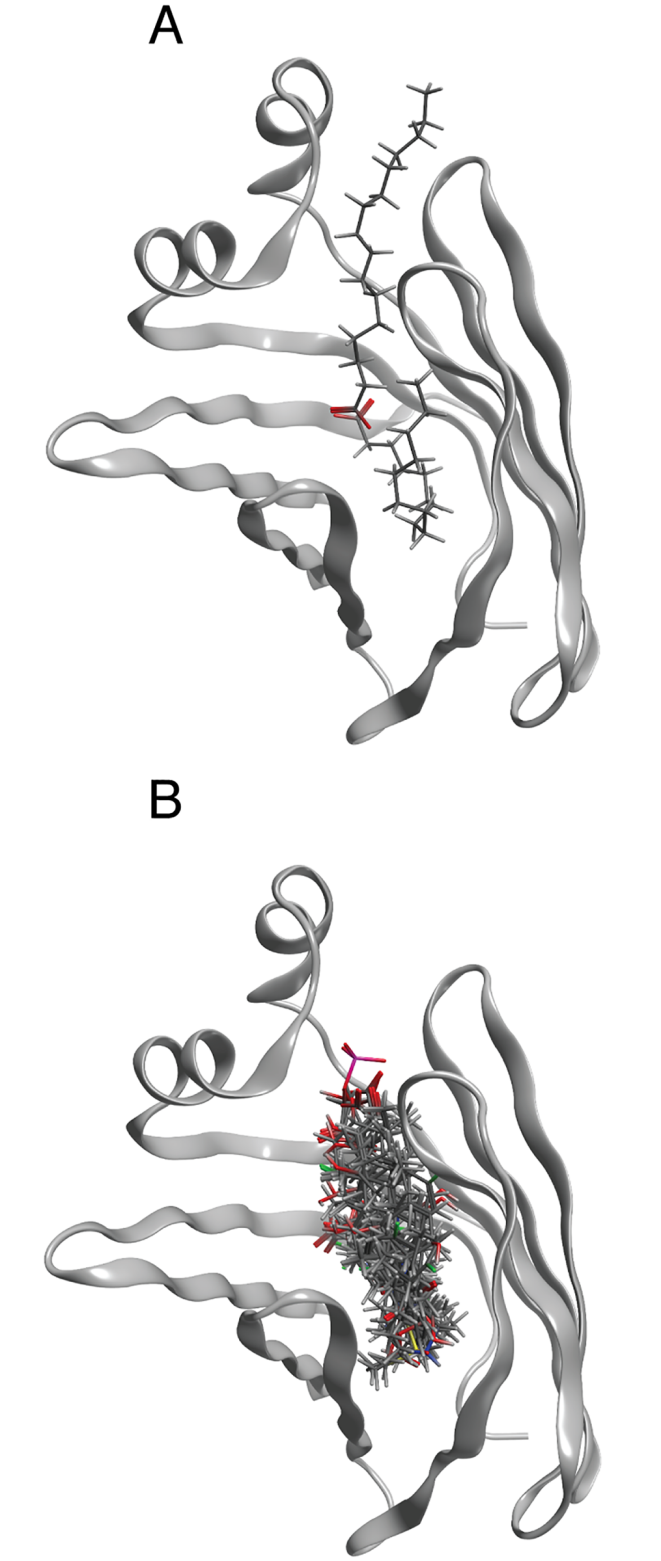
Panel A: *Holo* h-FABPL crystallized to contain two palmitate molecules (pdb code: 3STK). Panel B: h-FABPL with all the test ligands docked in its cavity.

The rigid receptor protocol we have used in all the docking procedures does not permit any movement of the protein parts during the placement steps; rearrangements are instead allowed to occur during the *ligX-optimization* step for affinity evaluation. However, since we used as target the structure of the *holo* protein, the shape of the calyx was already rearranged and optimized for binding in all parts of the cavity. Affinity of FABPLs for fatty acids scores one-order-of-magnitude higher with the inward than with the outward ligand (K_d_, of h-FABPL for oleic acid, 0.26 *vs* 5 μM [57]). Indeed, as shown by Fig 11, the position of all the docked ligands largely overlaps with that of the inward fatty acid in the crystallized *holo* structure. This evidence suggests that the estimated affinities are biased, by the inherent approximation of the procedure, to a similar extent with FABPL (human and chicken) as with any of the other FABPs. We did not actually assess whether a second molecule of each ligand could be docked after the first one, and with which affinity. On purely geometrical grounds, binding of a second molecule seemed possible in all cases.

In addition to the above possible implications in the clinical use of the test drugs, the outcome of our investigation is a better characterization of the interaction repertoire of intracellular calycins, and the recognition of which amino acids and which (permanent) water molecules are more often involved in interactions. As for ligands, the apolar character has been confirmed as one determinant of the affinity, possibly the single most relevant among manifold factors.

## Supporting Information

S1 Fig Sequence alignment for the selected calycins (http://www.ebi.ac.uk/Tools/msa/tcoffee/). Color-coding by similarity according to BLOSUM62.
(TIF)

S2 Fig Validation of the docking protocol. Panel A: Superposition of crystallographic (C atoms in dark gray) and docked palmitate (C atoms in green) in 2HNX structure. Panel B: Superposition of crystallographic (C atoms in dark gray) and docked ibuprofen (C atoms in green) in 6P6G structure. Panel C: Superposition of docked ibuprofen (C atoms in green) in 2HNX structure and crystallographic ibuprofen (C atoms in dark gray) in 6P6G structure, after alignment of the protein backbones.
(TIF)

S3 Fig ^19^F-NMR spectra recorded after the interaction of c-FABPL with 2-fluoropalmitate. This molecule is insoluble in aqueous buffers and reference data, reported in Table 3, were obtained in CDCl_3_. c-FABPL is able to solubilize this molecule: the occurrence of binding is strongly supported by the longitudinal relaxation time of both the observed resonances, which is one order of magnitude shorter than the one of the free ligand (0.24 and 0.23 s *vs* 2.47 s, Table 3), and by their negative n.O.e.’s (η = -0.54 and -0.58 *vs* 0.29, Table 3). Linewidth of the two signals 25 and 17 Hz, respectively. Operating conditions: PBS, 7 T, 298 K.
(TIF)

S4 Fig Protein ligand interaction fingerprints weighed via the PLIF procedure on the docking results for the 20 test ligands (Table 1) on each of the 7 test proteins (Table 2), both in the presence of consensus water (top panels) and without water (bottom panels). Just the latter condition applies to gastrotropin, for whose structure only NMR data are currently available. Water molecules relevant in interactions are boxed.
(TIF)

S5 Fig Log_10_K_i_ for the interaction of each of the test ligands (in Table 1) with c-FABPL and with the six human proteins with closest similarity to it (listed in Table 2; the caption to each panel contains the protein short name and the pdb code of the test structure). The *holo* structure of each protein was taken as *receptor* and ligand atoms as *docking site*; the procedure was repeated in the presence (dark grey bars) and in the absence (light grey bars) of consensus water molecules (Fig 1 and S2 Table).
(TIF)

S6 Fig Rendering of the items taking interaction with the test ligand in ≥ 30% of cases according to PLIF outputs (S4 Fig; these items are also listed in S2 Table). Color-coding of the test calycins by chain. The positions, at which a number of interacting amino acids cluster, correspond to: 16–19 = Phe16 of h-FABPH and of h-FABPA, Phe19 of h-FABPE; 51–56 = Ser51 of c-FABPL, Ile52 of h-FABPL, Ser53 of h-FABPA, Thr56 of h-FABPE, Thr53 of h-RETI; 78 = Arg78 of h-FABPH and of h-FABPA; 102–109 = Thr102 of h-FABPL, Arg 106 of h-FABPH and of h-FABPA, Arg109 of h-FABPE; 120–126 = Arg120 of c-FABPL, Arg122 of h-FABPL, Arg126 of h-FABPA.
(TIF)

S1 Table List of consensus water for the 11 calycin structures in Table 2 resolved by X-ray crystallography. (DOCX)

S2 Table Data from PLIF outputs (S4 Fig). (DOCX)

## References

[1] Ghuman J, Zunszain PA, Petitpas I, Bhattacharya AA, Otagiri M, Curry S. Structural basis of the drug-binding specificity of human serum albumin. J Mol Biol. 2005;353(1):38–52. 10.1016/j.jmb.2005.07.075 . 16169013

[2] Israili ZH, Dayton PG. Human alpha-1-glycoprotein and its interactions with drugs. Drug metabolism reviews. 2001;33(2):161–235. 10.1081/DMR-100104402 . 11495502

[3] Yamasaki K, Chuang VT, Maruyama T, Otagiri M. Albumin-drug interaction and its clinical implication. Biochim Biophys Acta. 2013;1830(12):5435–43. 10.1016/j.bbagen.2013.05.005 . 23665585

[4] Huang Z, Ung T. Effect of alpha-1-acid glycoprotein binding on pharmacokinetics and pharmacodynamics. Current drug metabolism. 2013;14(2):226–38. . 23092311

[5] Notarianni LJ. Plasma protein binding of drugs in pregnancy and in neonates. Clinical pharmacokinetics. 1990;18(1):20–36. . 2178848 10.2165/00003088-199018010-00002

[6] Gandhi M, Aweeka F, Greenblatt RM, Blaschke TF. Sex differences in pharmacokinetics and pharmacodynamics. Annual review of pharmacology and toxicology. 2004;44:499–523. 10.1146/annurev.pharmtox.44.101802.121453 . 14744256

[7] Soldin OP, Mattison DR. Sex differences in pharmacokinetics and pharmacodynamics. Clinical pharmacokinetics. 2009;48(3):143–57. 10.2165/00003088-200948030-00001 19385708 PMC3644551

[8] Barre J, Houin G, Brunner F, Bree F, Tillement JP. Disease-induced modifications of drug pharmacokinetics. International journal of clinical pharmacology research. 1983;3(4):215–26. . 6381335

[9] Kosa T, Maruyama T, Otagiri M. Species differences of serum albumins: I. Drug binding sites. Pharmaceutical research. 1997;14(11):1607–12. . 9434282 10.1023/a:1012138604016

[10] Matsumoto K, Sukimoto K, Nishi K, Maruyama T, Suenaga A, Otagiri M. Characterization of ligand binding sites on the alpha1-acid glycoprotein in humans, bovines and dogs. Drug metabolism and pharmacokinetics. 2002;17(4):300–6. . 15618681 10.2133/dmpk.17.300

[11] Krenzel ES, Chen Z, Hamilton JA. Correspondence of fatty acid and drug binding sites on human serum albumin: a two-dimensional nuclear magnetic resonance study. Biochemistry. 2013;52(9):1559–67. 10.1021/bi301458b . 23360066

[12] Fanali G, di Masi A, Trezza V, Marino M, Fasano M, Ascenzi P. Human serum albumin: from bench to bedside. Molecular aspects of medicine. 2012;33(3):209–90. 10.1016/j.mam.2011.12.002 . 22230555

[13] Takamura N, Maruyama T, Otagiri M. Effects of uremic toxins and fatty acids on serum protein binding of furosemide: possible mechanism of the binding defect in uremia. Clin Chem. 1997;43(12):2274–80. . 9439444

[14] Hackett MJ, Zaro JL, Shen WC, Guley PC, Cho MJ. Fatty acids as therapeutic auxiliaries for oral and parenteral formulations. Advanced drug delivery reviews. 2013;65(10):1331–9. 10.1016/j.addr.2012.07.012 22921839 PMC3537895

[15] Niwa T. Update of uremic toxin research by mass spectrometry. Mass spectrometry reviews. 2011;30(3):510–21. 10.1002/mas.20323 . 21328600

[16] Gambacorti-Passerini C, Barni R, le Coutre P, Zucchetti M, Cabrita G, Cleris L, et al Role of alpha1 acid glycoprotein in the in vivo resistance of human BCR-ABL(+) leukemic cells to the abl inhibitor STI571. J Natl Cancer Inst. 2000;92(20):1641–50. Epub 2000/10/19. . 11036109 10.1093/jnci/92.20.1641

[17] Zsila F, Bikadi Z, Malik D, Hari P, Pechan I, Berces A, et al Evaluation of drug-human serum albumin binding interactions with support vector machine aided online automated docking. Bioinformatics. 2011;27(13):1806–13. 10.1093/bioinformatics/btr284 . 21593135

[18] Vallianatou T, Lambrinidis G, Tsantili-Kakoulidou A. In silico prediction of human serum albumin binding for drug leads. Expert opinion on drug discovery. 2013;8(5):583–95. 10.1517/17460441.2013.777424 . 23461733

[19] Kratz F. Albumin as a drug carrier: design of prodrugs, drug conjugates and nanoparticles. Journal of controlled release: official journal of the Controlled Release Society. 2008;132(3):171–83. 10.1016/j.jconrel.2008.05.010 . 18582981

[20] Kratz F, Elsadek B. Clinical impact of serum proteins on drug delivery. Journal of controlled release: official journal of the Controlled Release Society. 2012;161(2):429–45. 10.1016/j.jconrel.2011.11.028 . 22155554

[21] Elzoghby AO, Samy WM, Elgindy NA. Albumin-based nanoparticles as potential controlled release drug delivery systems. Journal of controlled release: official journal of the Controlled Release Society. 2012;157(2):168–82. 10.1016/j.jconrel.2011.07.031 . 21839127

[22] Sleep D, Cameron J, Evans LR. Albumin as a versatile platform for drug half-life extension. Biochim Biophys Acta. 2013;1830(12):5526–34. 10.1016/j.bbagen.2013.04.023 . 23639804

[23] Flower DR, North ACT, Sansom CE. The lipocalin protein family: Structural and sequence overview. Biochym Biophys Acta. 2000;1482:9–24. 10.1016/s0167-4838(00)00148-5 11058743

[24] Åkerstrom B, Flower DR, Salier J-P. Lipocalins: Unity in diversity. Biochim Biophys Acta. 2000;1482:1–8. 11058742 10.1016/s0167-4838(00)00137-0

[25] Skerra A. Lipocalins as a scaffold. Biochim Biophys Acta. 2000;1482(1–2):337–50. . 11058774 10.1016/s0167-4838(00)00145-x

[26] Weiss GA, Lowman HB. Anticalins versus antibodies: made-to-order binding proteins for small molecules. Chemistry & biology. 2000;7(8):R177–84. . 11048945 10.1016/s1074-5521(00)00016-8

[27] Skerra A. Alternative binding proteins: anticalins—harnessing the structural plasticity of the lipocalin ligand pocket to engineer novel binding activities. FEBS J. 2008;275(11):2677–83. 10.1111/j.1742-4658.2008.06439.x . 18435758

[28] Richter A, Eggenstein E, Skerra A. Anticalins: exploiting a non-Ig scaffold with hypervariable loops for the engineering of binding proteins. FEBS Lett. 2014;588(2):213–8. 10.1016/j.febslet.2013.11.006 . 24239535

[29] Schiefner A, Skerra A. The Menagerie of Human Lipocalins: A Natural Protein Scaffold for Molecular Recognition of Physiological Compounds. Accounts of chemical research. 2015 10.1021/ar5003973 . 25756749

[30] Eberini I, Fantucci P, Guerini Rocco A, Gianazza E, Galluccio L, Maggioni D, et al Computational and experimental approaches for assessing the interactions between the model calycin β-lactoglobulin and two antibacterial fluoroquinolones. Proteins. 2006;65:555–67. 17001652 10.1002/prot.21109

[31] Eberini I, Rocco AG, Mantegazza M, Gianazza E, Baroni A, Vilardo MC, et al Computational and experimental approaches assess the interactions between bovine beta-lactoglobulin and synthetic compounds of pharmacological interest. Journal of molecular graphics & modelling. 2008;26(6):1004–13. . 17905618 10.1016/j.jmgm.2007.08.006

[32] Barbiroli A, Beringhelli T, Bonomi F, Donghi D, Ferranti P, Galliano M, et al Bovine beta-lactoglobulin acts as an acid-resistant drug carrier by exploiting its diverse binding regions. Biol Chem. 2010;391(1):21–32. Epub 2009/11/19. 10.1515/BC.2010.008 . 19919177

[33] Scapin G, Spadon P, Pengo L, Mammi M, Zanotti G, Monaco HL. Chicken liver basic fatty acid-binding protein (pI = 9.0): Purification, crystallization and preliminary X-ray data. FEBS Lett. 1988;240:196–200. 3191992 10.1016/0014-5793(88)80367-3

[34] Perduca M, Bossi A, Goldoni L, Monaco HL, Righetti PG. Crystallization of chicken liver (basic) fatty acid binding protein after purification in multicompartment electrolyzers with isoelectric membranes. Electrophoresis. 2000;21:2316–20. 10939440 10.1002/1522-2683(20000701)21:12<2316::aid-elps2316>3.0.co;2-0

[35] Ross GA, Morris GM, Biggin PC. Rapid and accurate prediction and scoring of water molecules in protein binding sites. PLoS ONE. 2012;7(3):e32036 10.1371/journal.pone.0032036 22396746 PMC3291545

[36] Schievano E, Quarzago D, Spadon P, Monaco HL, Zanotti G, Peggion E. Conformational and binding properties of chicken liver basic fatty acid binding protein in solution. Biopolymers. 1994;34:879–87. 8054470 10.1002/bip.360340707

[37] Beringhelli T, Goldoni L, Capaldi S, Bossi A, Perduca M, Monaco HL. Interaction of chicken liver basic fatty acid-binding protein with fatty acids: A ^13^C NMR and fluorescence study. Biochemistry. 2001;40:12604–11. 11601984 10.1021/bi011009w

[38] Nichesola D, Perduca M, Capaldi S, Carrizo ME, Righetti PG, Monaco HL. Crystal structure of chicken liver basic fatty acid-binding protein complexed with cholic acid. Biochemistry. 2004;43(44):14072–9. Epub 2004/11/03. 10.1021/bi0489661 . 15518556

[39] Eberini I, Guerini Rocco A, Ientile AR, Baptista AM, Gianazza E, Tomaselli S, et al Conformational and dynamics changes induced by bile acids binding to chicken liver bile acid binding protein. Proteins. 2008;71(4):1889–98. Epub 2008/01/05. 10.1002/prot.21875 . 18175325

[40] Ricchiuto P, Rocco AG, Gianazza E, Corrada D, Beringhelli T, Eberini I. Structural and dynamic roles of permanent water molecules in ligand molecular recognition by chicken liver bile acid binding protein. J Mol Recognit. 2008;21(5):348–54. Epub 2008/07/26. 10.1002/jmr.908 . 18654997

[41] Tomaselli S, Ragona L, Zetta L, Assfalg M, Ferranti P, Longhi R, et al NMR-based modeling and binding studies of a ternary complex between chicken liver bile acid binding protein and bile acids. Proteins. 2007;69(1):177–91. 10.1002/prot.21517 . 17607743

[42] Tochtrop GP, Richter K, Tang C, Toner JJ, Covey DF, Cistola DP. Energetics by NMR: site-specific binding in a positively cooperative system. Proc Natl Acad Sci U S A. 2002;99(4):1847–52. 10.1073/pnas.012379199 11854486 PMC122282

[43] Capaldi S, Saccomani G, Fessas D, Signorelli M, Perduca M, Monaco HL. The X-ray structure of zebrafish (Danio rerio) ileal bile acid-binding protein reveals the presence of binding sites on the surface of the protein molecule. J Mol Biol. 2009;385(1):99–116. 10.1016/j.jmb.2008.10.007 . 18952094

[44] Guariento M, Assfalg M, Zanzoni S, Fessas D, Longhi R, Molinari H. Chicken ileal bile-acid-binding protein: a promising target of investigation to understand binding co-operativity across the protein family. Biochem J. 2010;425(2):413–24. 10.1042/BJ20091209 . 19874274

[45] Bellosta S, Paoletti R, Corsini A. Safety of statins: focus on clinical pharmacokinetics and drug interactions. Circulation. 2004;109(23 Suppl 1):III50–7. 10.1161/01.CIR.0000131519.15067.1f . 15198967

[46] Smathers RL, Petersen DR. The human fatty acid-binding protein family: evolutionary divergences and functions. Human genomics. 2011;5(3):170–91. 21504868 10.1186/1479-7364-5-3-170 PMC3500171

[47] Storch J, Thumser AE. Tissue-specific functions in the fatty acid-binding protein family. J Biol Chem. 2010;285(43):32679–83. 10.1074/jbc.R110.135210 20716527 PMC2963392

[48] Assfalg M, Gianolio E, Zanzoni S, Tomaselli S, Russo VL, Cabella C, et al NMR structural studies of the supramolecular adducts between a liver cytosolic bile acid binding protein and gadolinium(III)-chelates bearing bile acids residues: molecular determinants of the binding of a hepatospecific magnetic resonance imaging contrast agent. J Med Chem. 2007;50(22):5257–68. 10.1021/jm070397i . 17915850

[49] Tomaselli S, Zanzoni S, Ragona L, Gianolio E, Aime S, Assfalg M, et al Solution structure of the supramolecular adduct between a liver cytosolic bile acid binding protein and a bile acid-based gadolinium(III)-chelate, a potential hepatospecific magnetic resonance imaging contrast agent. J Med Chem. 2008;51(21):6782–92. 10.1021/jm800820b . 18939814

[50] Maeda K, Cao H, Kono K, Gorgun CZ, Furuhashi M, Uysal KT, et al Adipocyte/macrophage fatty acid binding proteins control integrated metabolic responses in obesity and diabetes. Cell metabolism. 2005;1(2):107–19. 10.1016/j.cmet.2004.12.008 . 16054052

[51] Sulsky R, Magnin DR, Huang Y, Simpkins L, Taunk P, Patel M, et al Potent and selective biphenyl azole inhibitors of adipocyte fatty acid binding protein (aFABP). Bioorg Med Chem Lett. 2007;17(12):3511–5. 10.1016/j.bmcl.2006.12.044 . 17502136

[52] Lan H, Cheng CC, Kowalski TJ, Pang L, Shan L, Chuang CC, et al Small-molecule inhibitors of FABP4/5 ameliorate dyslipidemia but not insulin resistance in mice with diet-induced obesity. J Lipid Res. 2011;52(4):646–56. 10.1194/jlr.M012757 21296956 PMC3284158

[53] Zhang M, Zhu W, Li Y. Small molecule inhibitors of human adipocyte fatty acid binding protein (FABP4). Medicinal chemistry. 2014;10(4):339–47. . 24024500 10.2174/15734064113096660045

[54] Chuang S, Velkov T, Horne J, Porter CJ, Scanlon MJ. Characterization of the drug binding specificity of rat liver fatty acid binding protein. J Med Chem. 2008;51(13):3755–64. 10.1021/jm701192w . 18533710

[55] He Y, Yang X, Wang H, Estephan R, Francis F, Kodukula S, et al Solution-state molecular structure of apo and oleate-liganded liver fatty acid-binding protein. Biochemistry. 2007;46(44):12543–56. 10.1021/bi701092r . 17927211

[56] He Y, Estephan R, Yang X, Vela A, Wang H, Bernard C, et al A nuclear magnetic resonance-based structural rationale for contrasting stoichiometry and ligand binding site(s) in fatty acid-binding proteins. Biochemistry. 2011;50(8):1283–95. 10.1021/bi101307h 21226535 PMC3072248

[57] Sharma A, Sharma A. Fatty acid induced remodeling within the human liver fatty acid-binding protein. J Biol Chem. 2011;286(36):31924–8. 10.1074/jbc.M111.270165 21757748 PMC3173104

