# Supplementary figures and images for: All-Purpose Containers? Lipid-Binding Protein – Drug Interactions
Source: PLoS One. 2015 Jul 13;10(7):e0132096. doi: 10.1371/journal.pone.0132096 (PMC4500398; doi:10.1371/journal.pone.0132096)

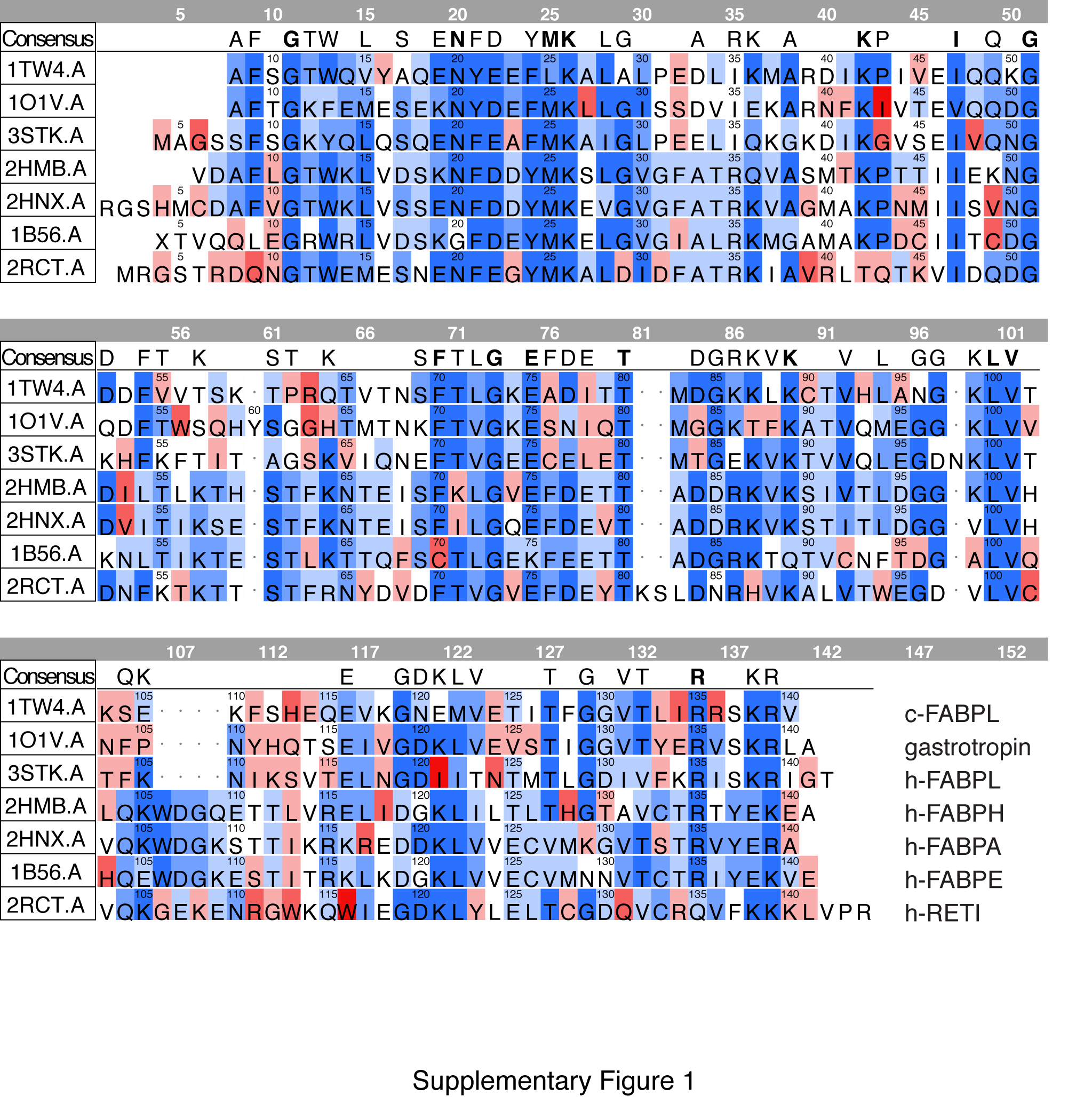

Supplement: S1 Fig — Color-coding by similarity according to BLOSUM62. (TIF) [file pone.0132096.s001.tif]

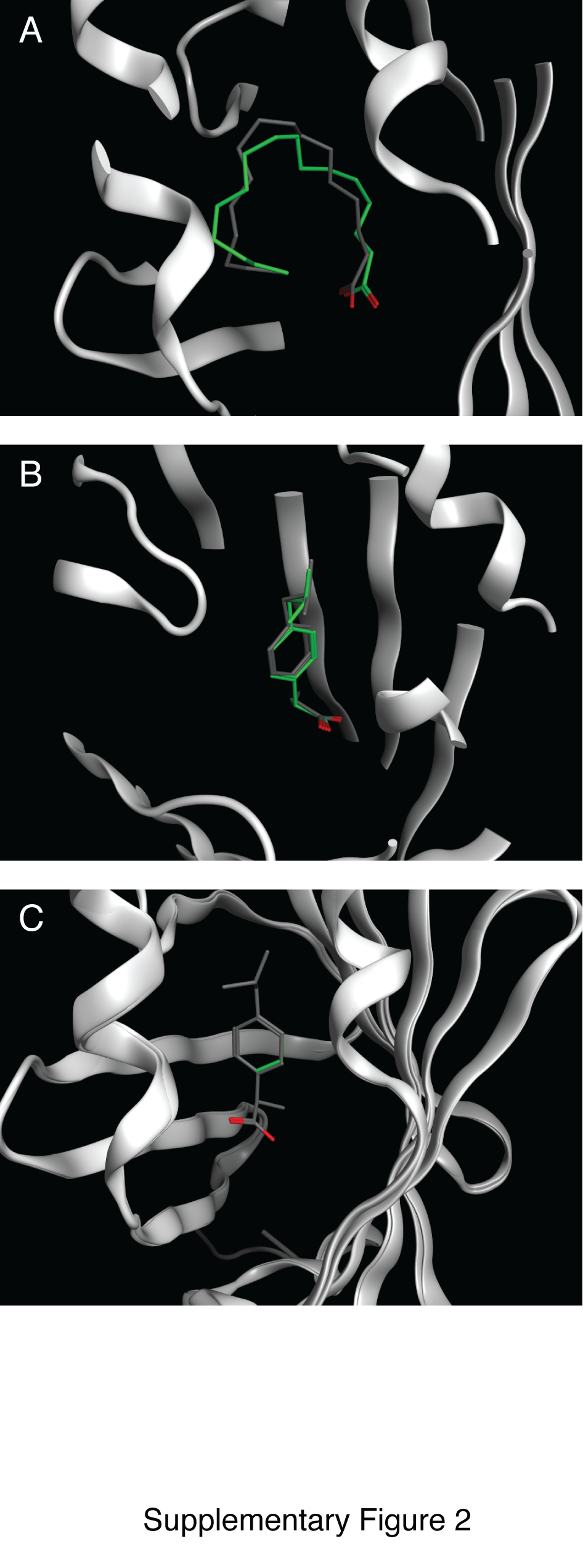

Supplement: S2 Fig — Panel A: Superposition of crystallographic (C atoms in dark gray) and docked palmitate (C atoms in green) in 2HNX structure. Panel B: Superposition of crystallographic (C atoms in dark gray) and docked ibuprofen (C atoms in green) in 6P6G structure. Panel C: Superposition of docked ibuprofen (C atoms in green) in 2HNX structure and crystallographic ibuprofen (C atoms in dark gray) in 6P6G structure, after alignment of the protein backbones. (TIF) [file pone.0132096.s002.tif]

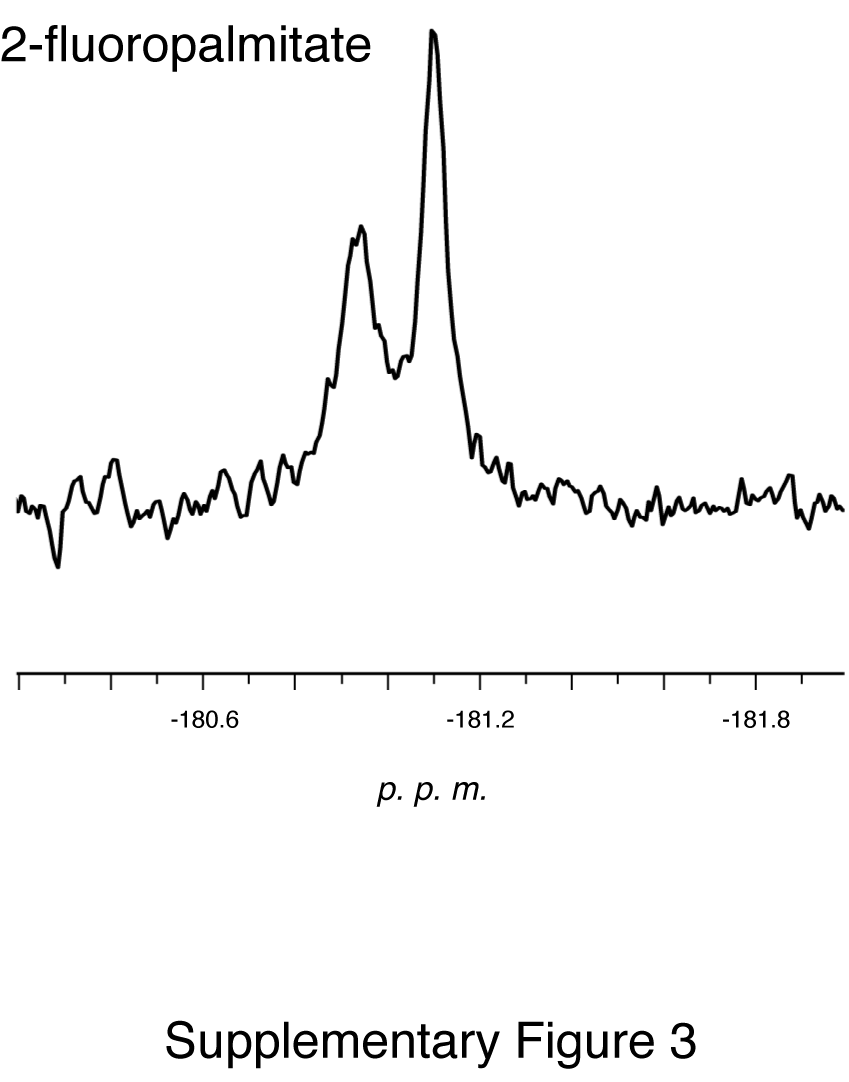

Supplement: S3 Fig — This molecule is insoluble in aqueous buffers and reference data, reported in Table 3, were obtained in CDCl3. c-FABPL is able to solubilize this molecule: the occurrence of binding is strongly supported by the longitudinal relaxation time of both the observed resonances, which is one order of magnitude shorter than the one of the free ligand (0.24 and 0.23 s vs 2.47 s, Table 3), and by their negative n.O.e.’s (η = -0.54 and -0.58 vs 0.29, Table 3). Linewidth of the two signals 25 and 17 Hz, respectively. Operating conditions: PBS, 7 T, 298 K. (TIF) [file pone.0132096.s003.tif]

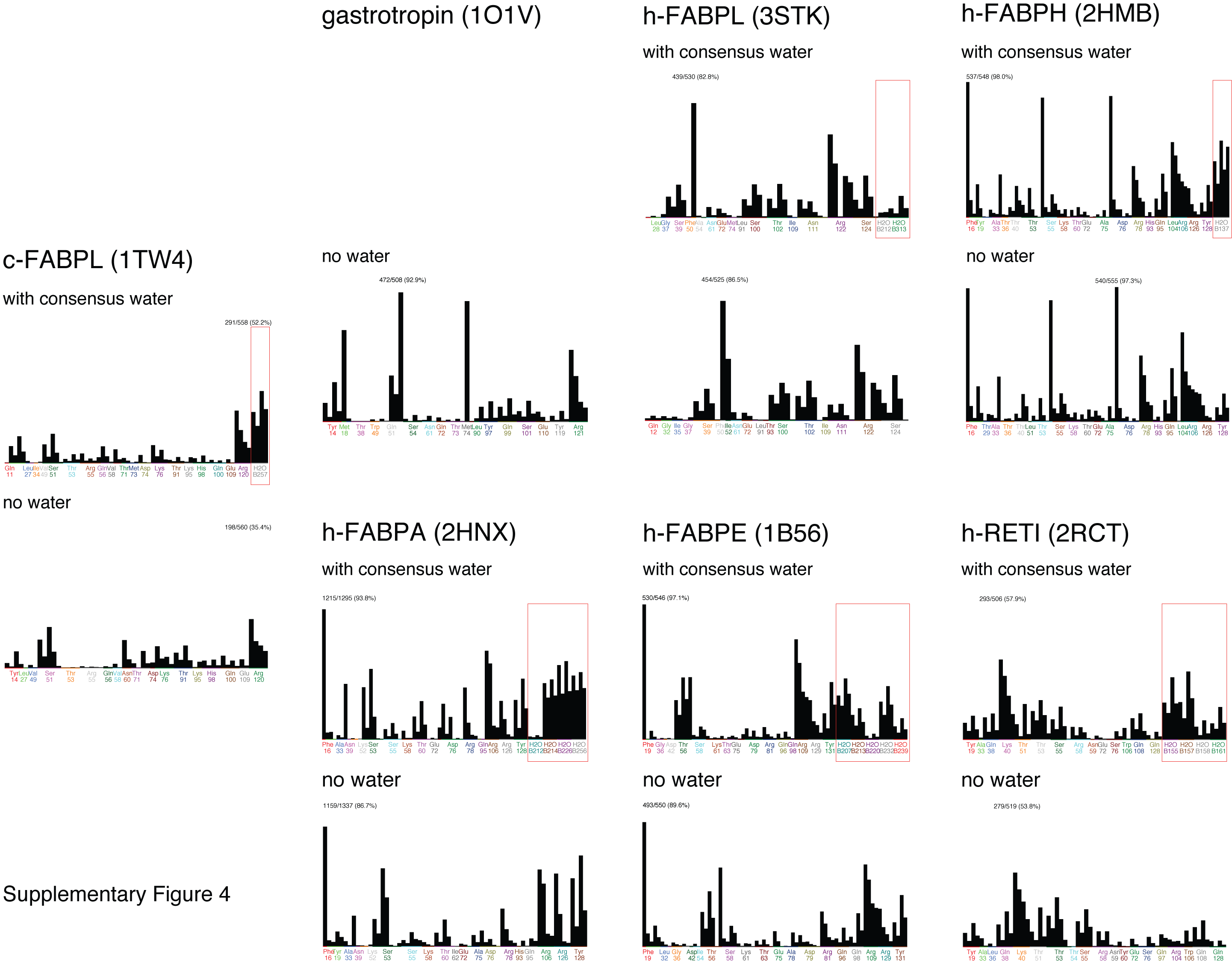

Supplement: S4 Fig — Just the latter condition applies to gastrotropin, for whose structure only NMR data are currently available. Water molecules relevant in interactions are boxed. (TIF) [file pone.0132096.s004.tif]

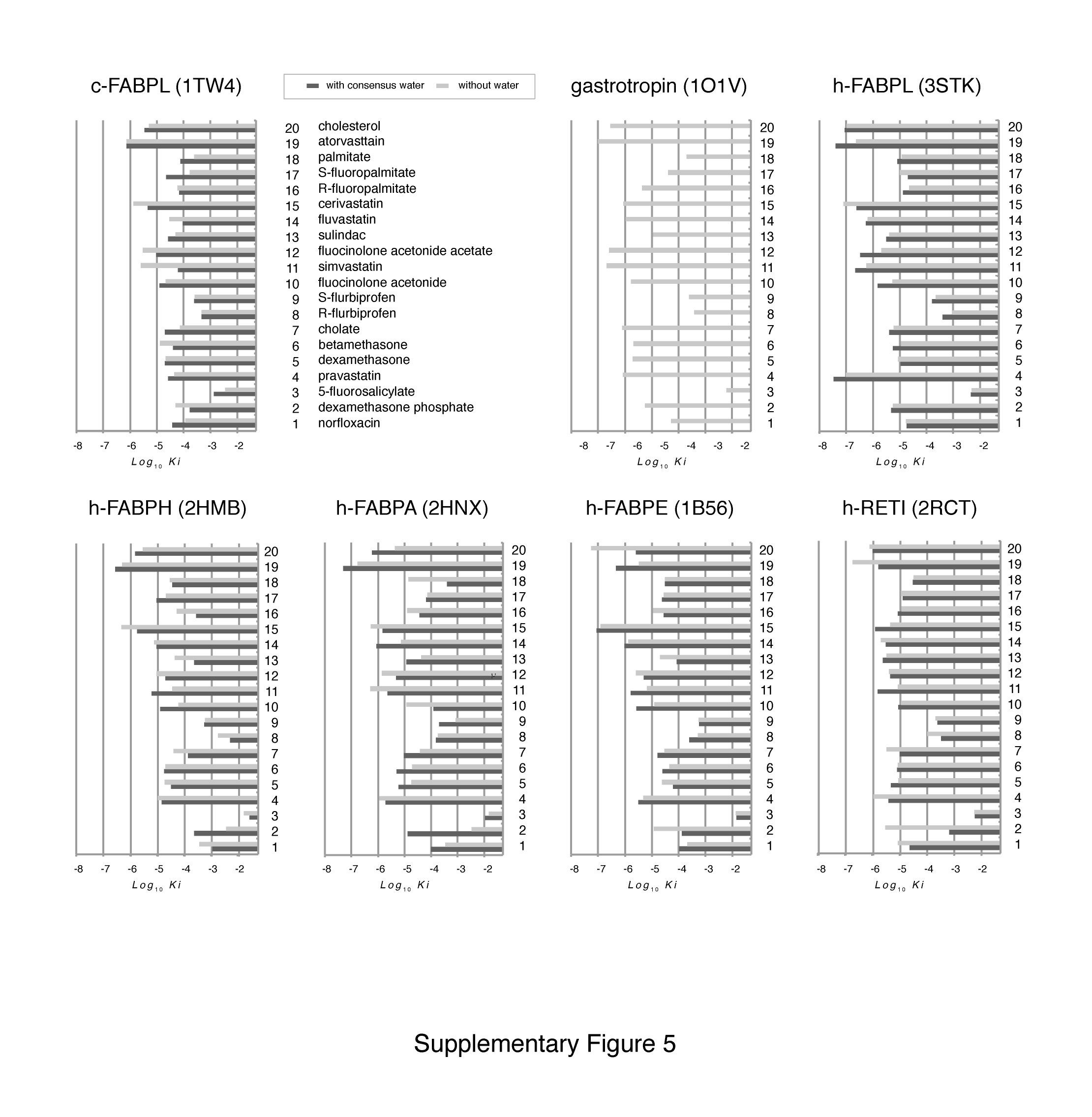

Supplement: S5 Fig — The holo structure of each protein was taken as receptor and ligand atoms as docking site; the procedure was repeated in the presence (dark grey bars) and in the absence (light grey bars) of consensus water molecules (Fig 1 and S2 Table). (TIF) [file pone.0132096.s005.tif]

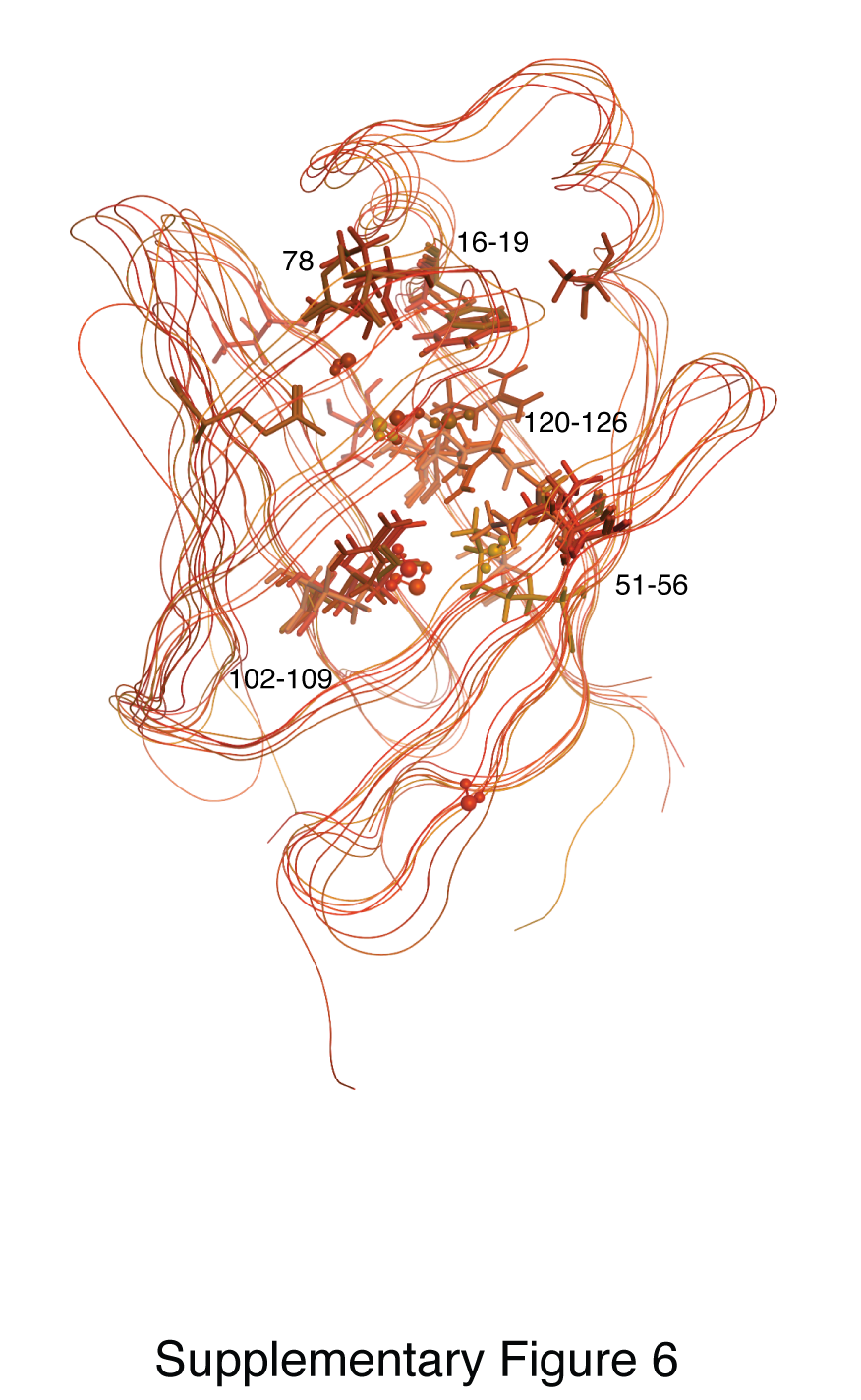

Supplement: S6 Fig — Color-coding of the test calycins by chain. The positions, at which a number of interacting amino acids cluster, correspond to: 16–19 = Phe16 of h-FABPH and of h-FABPA, Phe19 of h-FABPE; 51–56 = Ser51 of c-FABPL, Ile52 of h-FABPL, Ser53 of h-FABPA, Thr56 of h-FABPE, Thr53 of h-RETI; 78 = Arg78 of h-FABPH and of h-FABPA; 102–109 = Thr102 of h-FABPL, Arg 106 of h-FABPH and of h-FABPA, Arg109 of h-FABPE; 120–126 = Arg120 of c-FABPL, Arg122 of h-FABPL, Arg126 of h-FABPA. (TIF) [file pone.0132096.s006.tif]
