# Supplementary material for: All-Purpose Containers? Lipid-Binding Protein – Drug Interactions
Source: PLoS One. 2015 Jul 13;10(7):e0132096. doi: 10.1371/journal.pone.0132096 (PMC4500398; doi:10.1371/journal.pone.0132096)
Supplement: S1 Table — (DOCX) [file pone.0132096.s007.docx]

**S1 Table. List of consensus water for the 11 calycin structures in Table 2 resolved by X-ray crystallography.**

| **pdb code** | **water molecule i.d.** | | | | | | | | | | | | | | | | | | |
| --- | --- | --- | --- | --- | --- | --- | --- | --- | --- | --- | --- | --- | --- | --- | --- | --- | --- | --- | --- |
| **(1TW4)** | 34 | 23 | 38 | 97 | 25 | 54 | 59 | 20 | 21 | 60 | 65 | 109 | 88 | 98 | 19 |  |  |  |  |
| **(1B56)** | 36 | 10 | 16 | 20 | 28 | 38 | 13 | 39 | 31 | 7 | 6 |  |  |  |  |  |  |  |  |
| **(1TVQ)** | 8 | 3 | 43 | 24 | 1 | 36 | 11 | 1 | 75 | 117 | 113 | 12 | 5 | 76 |  |  |  |  |  |
| **(2HMB)** | 46 | 17 | 11 | 31 | 4 | 8 | 13 |  |  |  |  |  |  |  |  |  |  |  |  |
| **(2HNX)** | 54 | 1 | 22 | 12 | 8 | 14 | 3 | 34 | 56 | 26 | 17 | 19 | 20 | 15 | 48 | 50 | 102 | 13 |  |
| **(2RCQ)** | 127 | 92 | 9 | 3 | 22 | 5 | 137 | 42 | 27 | 57 | 43 | 33 | 97 | 36 | 58 | 120 | 19 | 67 |  |
| **(2RCT)** | 54 | 13 | 9 | 3 | 6 | 15 | 5 | 122 | 51 | 43 | 62 | 27 | 21 | 35 | 45 | 77 | 29 | 46 |  |
| **(3RZY)** | 27 | 9 | 47 | 59 | 32 | 38 | 51 | 13 | 118 | 102 | 81 | 54 | 60 | 49 | 35 | 85 | 75 | 109 | 41 |
| **(3STK)** | 114 | 94 | 126 | 182 | 100 | 81 | 101 | 102 | 140 | 1 | 129 | 103 | 151 | 154 | 128 | 116 | 152 | 125 |  |
| **(3STN)** | 2 | 7 | 12 | 6 |  |  |  |  |  |  |  |  |  |  |  |  |  |  |  |
| **(4LKP)** | 65 | 7 | 14 | 28 | 31 | 15 | 3 | 20 | 38 | 17 | 60 | 94 | 62 | 51 | 26 | 55 | 16 |  |  |
