# Supplementary material for: All-Purpose Containers? Lipid-Binding Protein – Drug Interactions
Source: PLoS One. 2015 Jul 13;10(7):e0132096. doi: 10.1371/journal.pone.0132096 (PMC4500398; doi:10.1371/journal.pone.0132096)
Supplement: S2 Table — (DOCX) [file pone.0132096.s008.docx]

**S2 Table. Data from PLIF outputs (S3 Figure).**

| **protein** | **water** | **protein-ligand interactions with occurrence ≥ 30%** |
| --- | --- | --- |
| **1TW4** | + | Arg 120; B257 |
|  | - | Ser 51; Arg 120 |
| **gastrotropin** | - | Met 18; Gln 51; Met 74; Arg 121 |
| **3STK** | + | Phe 50; Arg 122; Ser 124 |
|  | - | Phe 50; Ile 52; Thr 102; Arg 122; Ser 124 |
| **2HMB** | + | Phe 16; Ala 33; Thr 53; Ala 75; Arg 78; Gln 95; Leu 104; Arg 106; B137 |
|  | - | Phe 16; Ala 33; Thr 53; Ala 75; Arg 78; Leu 104; Arg 106 |
| **2HNX** | + | Phe 16; Ala 33; Ser 53; Arg 78; Arg 106; Tyr 128; B214; B226; B256 |
|  | - | Phe 16; Ser 53; Arg 106; Arg 126; Tyr 128 |
| **1B56** | + | Phe 19; Thr 56; Arg 109, Tyr 131; B207; B232 |
|  | - | Phe 19: Thr 56; Arg 109; Tyr 131 |
| **2RCT** | + | Lys 40; B155; B157 |
|  | - | Lys 40; Thr 53 |
